# Review of the Most Important Research Trends in Potential Chemotherapeutics Based on Coordination Compounds of Ruthenium, Rhodium and Iridium

**DOI:** 10.3390/ph18111728

**Published:** 2025-11-13

**Authors:** Agnieszka Gilewska, Barbara Barszcz, Joanna Masternak

**Affiliations:** Institute of Chemistry, Jan Kochanowski University in Kielce, Uniwersytecka 7, 25-406 Kielce, Poland; barbara.barszcz@ujk.edu.pl

**Keywords:** metal Ru, Rh, Ir complexes, classes of complexes, potential chemotherapeutics, photosensitizers of PDT, PACT

## Abstract

This review paper presents a comprehensive literature analysis that elucidates the global engagement of research teams in addressing the important problem of finding effective oncology drugs based on the following platinum group metal ions: ruthenium, rhodium and iridium. The necessity to search for new drugs can be attributed, in part, to the predominance of platinum-based chemotherapeutics in clinical practice. However, these drugs face limitations in their clinical application due to their inherent toxicity and the development of resistance by cancer cells. A distinctive attribute of these metal compounds is the formation of diamagnetic stable complexes on +II (Ru) and +III (Rh, Ir) oxidation degrees with a d^6^ electron configuration, a coordination number of six and an octahedral or pseudo-octahedral structure. In this paper we have systematised the findings presented in the literature by classifying the most significant categories of ruthenium, rhodium and iridium compounds, namely piano-stool-type arenes, polypyridine and cyclometalated complexes, dimers and multinuclear complexes. Additionally, the most crucial research challenges connected with metal complexes that have been addressed by scientists have been presented: (i) the application of prodrugs in cancer therapy; (ii) the deployment of complexes as sensitizers in PDT and PACT; (iii) the exploration of complexes as inhibitors of enzymes and biocatalysts; and (iv) the investigation of multiple-target complexes. Furthermore, the objective was to emphasise the accomplishments in this domain in recent years by identifying compounds that have entered the clinical trial phase.

## 1. Introduction

Contemporary medical problems in cancer treatment have led to intensive exploration of the synthesis, and physicochemical and biological properties, of platinum group metal complexes. Numerous interesting reviews have been written on Ru, Rh, and Ir complexes [1,2,3,4,5,6,7,8,9,10,11,12]. After analysing the subject matter of the aforementioned papers, we concluded that it would be relevant and of interest to the reader to present a review paper that systematises the results of the biological studies to date by (i) characterising the most important classes of ruthenium, rhodium and iridium compounds; (ii) presenting the main research problems that have been addressed by scientists on the basis of these compounds; and (iii) describing the attempts to solve these problems in the search for new chemotherapeutic agents based on these platinum group metals.

At the same time, we wish to emphasise that it will not be possible to realise the goals set in this work without recourse to classical knowledge of the coordination chemistry of metals. This knowledge is also necessary to understand the biological processes in which the studied complexes participate. Therefore, the introduction will cover the significance of central ions and ligands in the design of potential chemotherapeutics, as well as reviewing interesting methods for synthesising metal complexes with inert ions (Ru, Rh and Ir). Coordination chemistry, which includes the research on the synthesis, properties and uses of complex compounds, has developed dynamically over the last few decades [13]. Coordination compounds are of particular interest due to their diverse structures and interesting physicochemical properties, which give rise to a multitude of applications. Owing to the achievements of modern science, metal complexes are used in industry and in new fields of technology [14,15,16,17,18,19,20,21]. At the same time, coordination chemistry is undergoing intensive development in new interdisciplinary fields, such as the fields of bioinorganic, bioorganic and organometallic chemistry, to study topics including the synthesis of new anticancer drugs based on transition metal complexes. New chemotherapeutic agents are urgently needed in the fight against cancer. According to the WHO, cancer is already responsible for at least 13% of deaths worldwide, and it is estimated that by 2025, mortality will increase by approximately 20 million deaths per year [22,23]. The complexity of the treatment problem is mainly because cancers arise in different tissue types, and have multiple aetiologies and an infinite number of combinations of genetic or epigenetic alterations. Research has shown that metal complexes with organic ligands exhibit greater biological activity in the body than organic chemotherapeutics. This is partly because of the geometric possibilities of such complexes, which allow the modulation of steric properties to facilitate coordination with, for example, the donor atoms of biological targets (DNA and proteins) [24]. Moreover, the physicochemical diversity, including the redox diversity, supplied by a wide selection of metal ions and ligands, enables the maintenance of the proper electron balance in the organism and of kinetic properties tuned to the requirements of mitosis. Since Rosenberg’s discovery of cisplatin in 1965, metal-based complexes have been a valuable platform in the search for anticancer drugs. Cisplatin was approved as a medication by the FDA in 1978 [1,25]. Furthermore, this discovery established chemotherapy as one of the most significant methods employed by the medical community in the fight against cancer, alongside mastectomy, radiotherapy and hormone therapy, which are often used in combination. Currently, most anticancer drugs used in clinical practice are based on platinum: cisplatin, carboplatin and oxaliplatin are used globally, and nedaplatin (Japan), lobaplatin (China) and heptaplatin (South Korea) are used in specific locations [26,27]. However, the use of chemotherapeutic agents in cancer treatment to date has shown certain limits due to their lack of selectivity and toxic side effects (neuro, nephro and ototoxic effects, as well as adverse effects, on the gastrointestinal tract) and, primarily, the innate or acquired resistance of cancer cells to the administered chemotherapy drugs [28,29,30]. Therefore, in recent years, there has been an intensive search for new chemotherapeutic agents based on ruthenium, rhodium and iridium complexes that have less toxic side effects, are more selective and address the problem of resistance [31,32,33,34]. These metals belong to the d-block elements of the platinum group. They are kinetically inert, rare elements that were discovered between 1803 and 1807, and occur as impurities in ores of other metals, such as platinum. The oxidation state of metal ions in compounds can vary: ruthenium from +II to +VIII, and rhodium and iridium from +I to +VI. The most stable coordination compounds involve the +II (Ru) and +III (Rh, Ir) oxidation states and are diamagnetic complexes with a d^6^ electron configuration, a coordination number of six and an octahedral or pseudo-octahedral structure. In the case of the *4d*-(Ru, Rh) and *5d*-(Ir) electron block cations, in the octahedral field of the ligands, there is greater cleavage of the d orbitals (large values of Δ_oct._ parameter) and less electron repulsion; thus, the d electrons have a lower pairing energy, which is advantageous for the formation of low-spin complexes.

The increased interest in this group of compounds in the medical field is inspired, among other things, by (i) the kinetic parameters of ligand exchange, similar to those of cisplatin (rate comparable to the rate of cell division (mitosis)), (ii) low redox potentials, allowing different oxidation levels (II, III, IV), (iii) the ability to bind to proteins, facilitating transport, (iv) lower toxicity to healthy cells in the body and (v) the ability to mimic iron ions in the transport of the drug into the tumour cell, especially in the case of ruthenium complexes [35,36,37].

The resulting complexes’ biological activity is influenced by both the metal ions and the ligands. Therefore, researchers have focused on the selection of appropriate ligands that can form stable bonds in new compounds with potential anticancer properties. In addition, in vivo, metal ions mostly bind to ligands according to acid–base (HSAB) theory. Ru(II), Rh(II), Rh(III) and Ir(III) ions all belong to the so-called intermediate class of Lewis acids, which suggests that they will form stable bonds with similar, i.e., intermediate class, Lewis bases but also with bases belonging to both the hard and the soft classes of Lewis bases. Among these bases, organic compounds that satisfy the above criteria include N,N-, N,O-, N,S- and N,C-donor ligands that can form stable chelate rings.

**Figure 1:**
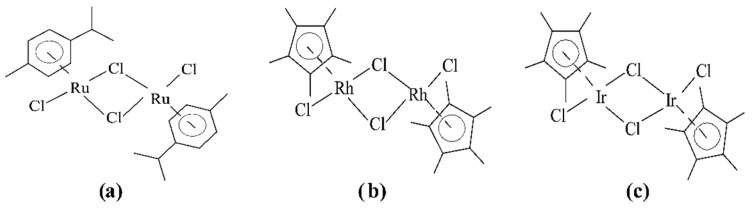
Schematic of structures of organometallic precursors: (**a**) [(η^6^-p-cymene)Ru(µ-Cl)Cl]_2_, (**b**) [CpRh(μ-Cl)Cl]_2_, (**c**) [CpIr(μ-Cl)Cl]_2_.The Ru(II) precursor thus contains p-cymene(4-isopropyltoluene), an aromatic ligand containing a six-membered arene ring, which contributes six π electrons to M–C bonds in complexes with metals. Among the sandwich complexes formed with transition metals, those with metals in the sixth group of the periodic table stand out in terms of stability. In contrast, the pentamethylcyclopentadienyl (Cp) found in the rhodium and iridium precursors is an anionic aromatic ligand that also contains six π electrons in the ring, according to Hückel’s rule. The ligand most commonly binds to metals in the η^5^-C_5_H_5_ form, and the molecular π orbitals of the five carbon atoms of the ligand Cp combine with the corresponding s, p and d orbitals of the metal ion. Additionally, a method using a suitable precursor in the form of the cyclometalated dimers of ruthenium, rhodium and iridium in the presence of selected N,N-, N,O- and N,S-donor ligands leads to the synthesis of another intensively studied group of complexes called cyclometalated complexes (for synthesis details, see the review in [38]).

The inert metal ion complexes of Ru, Rh and Ir require appropriate synthetic methods. A systematic review of the literature shows that three main synthetic routes for the preparation of ruthenium coordination compounds: the silver salt precipitation method, the precursor method and the mother solution method. For rhodium and iridium complexes, the most popular methods are, instead, the precursor method and the direct method using a hydrated metal chloride salt. For the synthesis of a large group of half-sandwich complexes of Ru(II), Ir(III) and Rh(III), dimeric precursors are used (Figure 1).

## 2. Potential Ruthenium-Based Chemotherapeutics: Key Research Trends

The coordination chemistry of ruthenium compounds has developed rapidly, and most reports on their use in medicine and pharmacology are concerned with investigations of their potential use as anticancer agents [36,37,39,40,41,42,43]. The main strengths of ruthenium complexes in this regard are their ease of ligand exchange in substitution reactions, their ability to change oxidation degrees under physiological conditions (in the range of +II and +III) and their ability to imitate iron in some biological processes. Mainly, Ru(II) complexes are more stable than Ru(III) complexes, and aquatation reaction can be regulated by changing the type of ligand and the charge of the complex [44]. Apart from their few side effects, the greatest advantage of synthesised ruthenium compounds is their cytotoxic activity and antimetastaticeffect [45]. Among the great structural diversity of ruthenium complexes with cytotoxic activity, three main classes of these compounds should be distinguished: (i) the prodrug group of Ru(III), (ii) the piano-stool compounds of Ru(II) and (iii) polypyridine and cyclometalated complexes. To systematise the existing knowledge, the problems arising in intensive research on the use of ruthenium coordination compounds in cancer therapy will be presented, with reference to the abovementioned classes of compounds.

### 2.1. Ru(III) Complexes as Prodrugs in Cancer Therapy

Ruthenium(III) complexes behave as prodrugs because they can be reduced to more active forms of Ru(II) in a process known as ‘activation by reduction’. This process takes place in cancer cells characterised hypoxia, acidic pH and a reducing environment (e.g., GSH, etc.) [46,47]. Furthermore, it has been speculated that ruthenium(III) complexes accumulate in cancer cells through a highly selective iron transport mechanism and albumin [48]. Thus, with regard to ruthenium complexes, the desired biological response can be triggered by the activation of the prodrug in vivo by changing the oxidation state of Ru(III) to Ru(II) as a result of in situ reduction reactions in the biological environment and/or via photochemical processes through the application of the appropriate ligand before reaching the target site [45].

**Figure 2:**
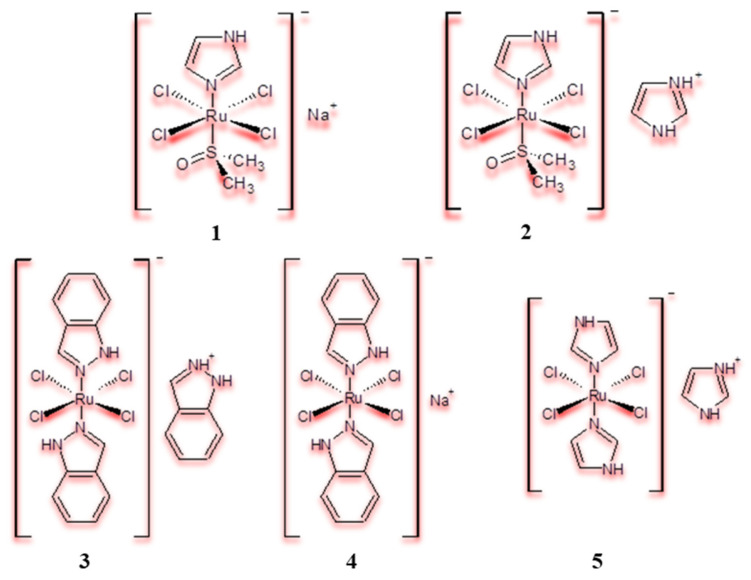
Schematic of structures of NAMI (**1**), NAMI-A (**2**), KP1019 (**3**), NKP1339 (**4**) and KP418 (**5**) [55].

In the field of ruthenium(III)-based chemotherapeutics from the prodrug group, a successful experiment by E. Alessio’s team [49,50,51,52] involved the development of the following complexes: NAMI and NAMI-A (Figure 2), which both showed weak in vitro antitumour activity but high antimetastatic activity in several leukaemia cell lines [53]. The acronym **NAMI** stands for “New Antitumour Metastasis Inhibitor”.

Accordingly, the NAMI-A complex was subjected to phase I clinical trials (1999) and received a positive evaluation [46,54,55]. Another group of potential prodrug chemotherapeutics based on Ru(III) compounds was developed by Keppler’s team [56,57,58,59,60]. One of the most promising compounds was KP1019 (see Figure 2), which ultimately passed phase I clinical trials. It demonstrated activity against cisplatin-resistant HCT, with low systemic toxicity [49].

However, owing to its limited solubility, KP1019 does not meet all the requirements for a drug, and its analogue was developed in the form of a sodium salt NKP1339 (Figure 2). This compound also entered phase I clinical trials after it demonstrated potent in vitro cytotoxic activity against cisplatin-resistant HCT cells [61,62]. Although the MoA of these ruthenium complexes is not defined, many research results confirm that the complexes interact with human proteins, which act as essential carriers in the transport of the chemotherapeutic agent to the tumour [63]. Research has demonstrated that the formation of adducts, protein–Ru(III) complexes, improves not only drug delivery but also drug selectivity, through the effect of enhanced retention and permeability (EPR). For example, macromolecules, such as albumin complexes, passively accumulate in solid tumours [64]. The process of reducing Ru(III) compounds to Ru(II) using biomolecules, such as GSH, cysteine or ascorbic acid, is also of particular therapeutic importance. As a result of this reduction, the Ru(II) complexes formed in situ are more labile and, therefore, more biologically active. In addition, cellular reducing agents and oxygen deficiency facilitate the entry of the resulting Ru(II) ions into the nucleus of cancer cells. In this sense, KP1019/KP1339 can be considered prodrugs activated by reduction reactions [65]. Recent studies have shown that the MoA of KP1019 and KP1339 also results from protein stress and the disruption of mechanisms for maintaining oxidative balance in the cell. Notably, the abovementioned NAMI-A has a different MoA from KP1019/KP1339. When comparing KP1019 to NAMI-A, the former exhibits a cytotoxic effect, while the latter exhibits an antimetastatic effect [66]. Furthermore, in 2011, interaction of the complex with RNA was revealed, suggesting that nucleic acids could be a biological target for this type of Ru(III) complex as well [67]. Interestingly, model studies comparing NAMI-A and KP1019 binding to tRNA have revealed that both Ru(III) compounds bind tightly to the oligonucleotide acid under investigation [68]. These results suggest that this interaction with RNA may induce ribotoxic effects, thereby affecting global protein synthesis [67].

### 2.2. Ruthenium(II) Complexes as Sensitisers in PDT and PACT Therapy

**Figure 3:**
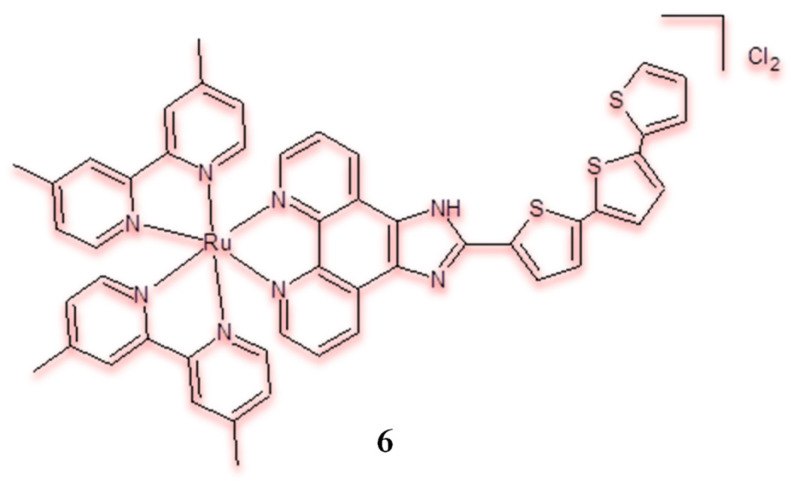
Schematic of structure of TLD-1433 [69].

Prodrugs based on ruthenium complexes also include polypyridine and cyclometalated Ru(II) complexes used as PSs in PDT and PACT. PSs should be considered prodrugs, as the actual therapeutic effect of PDT is due to ROS produced via light exposure. When complexes are used in PDT, these compounds generate ^1^O_2_ and other ROS, under the influence of light, which forms the basis of therapy by selectively targeting the cancer cells and tissues with the generated ROS (for details, see p. Ir). PDT is one of the least invasive methods for treating cancer, among other diseases, because the action of toxic singlet oxygen and other ROS is limited to the tumour, leaving healthy tissue alone and, thereby, eliminating the harmful side effects of platinum-based chemotherapies. Of particular note in this group of compounds is TLD-1433 (Figure 3), synthesised by S. A. McFarland et al. [69,70,71,72]. This compound is the only one of the rich group of Ru(II) complexes to have been directed to clinical trials. It has passed phase I trials in the treatment of bladder cancer and is currently being tested in phase II clinical trials. Preliminary data from a phase II study may support the use of light-activated Ru(II) complex PDT as a treatment option for patients with bacillus Calmette-Guérin (BCG)–unresponsive non–muscle invasive bladder cancer (NMIBC) carcinoma in situ (CIS) [73]. The compound exhibits selective activity and induces cancer cell death via ^1^O_2_ [74].

**Scheme 1:**
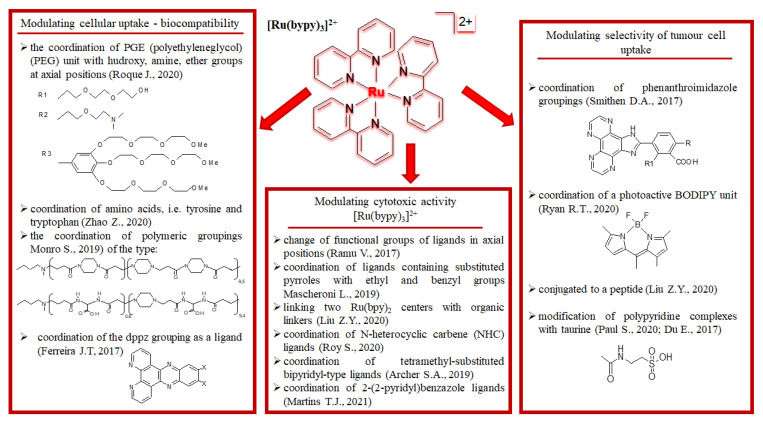
Model of complex [(Ru(bypy)_3_]^2+^ and its studied modifications [69,74,75,76,77,78,79,80,81,82,83,84,85,86].

Following the success of the polypyridyl TLD-1433 complex in clinical trials, researchers have turned their attention to the search for an ideal PS, as illustrated by the data shown in Scheme 1. Among other things, they have attempted to find a solution to the problems of (i) obtaining strong absorption in the range of 650–850 nm (‘phototherapeutic window’) to achieve better radiation penetration through human tissues; (ii) increasing the cellular uptake of a given PS by tuning its lipophilic/hydrophilic properties; and (iii) changing the substituents in the complex to increase the photosensitisation potential.

In this context, we refer readers to a very good review by Luca Conti and co-authors [4] for detailed information. The authors showed that the modifications mentioned above were aimed at improving (i) cellular uptake, (ii) biocompatibility, (iii) cytotoxic activity and (iv) selective tumour cell uptake (Scheme 1). The results presented [75,76,77,78,79,80,81,82,83,84,85,86,87,88] that the introduction of different substituents into the bipyridyl rings, or the replacement of bpy with a new ligand, modulates the hydrophilicity/lipophilicity and biocompatibility of the complexes. It has been shown that, in a series of complexes with an introduced PEG group, the hydrophilicity of the compounds decreases but at the same time the cytotoxicity of the chemotherapeutic agent against bladder cancer cells increases after exposure to red light [75]. The presence of polymeric groups increases the efficacy of PDT, especially in the case of cationic polymeric substituents, which form a macromolecular complex and, at the same time, have a high capacity to accumulate in the nucleus of cancer cells [76]. In contrast, halogen substituents in the [Ru(dppz-X_2_)_3_]^2+^ increase lipophilicity and reduce phototoxicity, despite increased cellular uptake. The induced effects were found to correlate with the value of the atomic radius of the halogen substituents [77]. Furthermore, the Ru(II) compounds obtained by A. Thompson and co-authors [78], which contain pyrrole ligands substituted with ethyl and benzyl groups, represent promising alternatives to traditional chemotherapeutics. The complexes obtained showed better cytotoxic properties than cisplatin against the HL-60 and SKMEL28 cancer cells. The cytotoxicity of this series of complexes depends on both the substituent in the pyrrole ring and the cell line used. On the other hand, studies by J. P. Selegue and co-authors [79] have shown that the introduction of N-heterocyclic carbenes (NHCs) instead of bpy tridonor ligands into the model Ru(II) complex has a favourable effect on the excited state lifetimes, efficient ^1^O_2_ production and photocytotoxicity of the ruthenium(II) compounds obtained. In turn, research conducted on modulating the selectivity of cancer cell uptake has shown an increase in selective phytotoxicity due to the introduction of phenanthroimidazole ligands [80], or a BODIPY unit [81] in the [Ru(bpy)_3_]^2+^ compound or substituents (taurine and peptides) [82,83] in the bpy ring.

**Figure 4:**
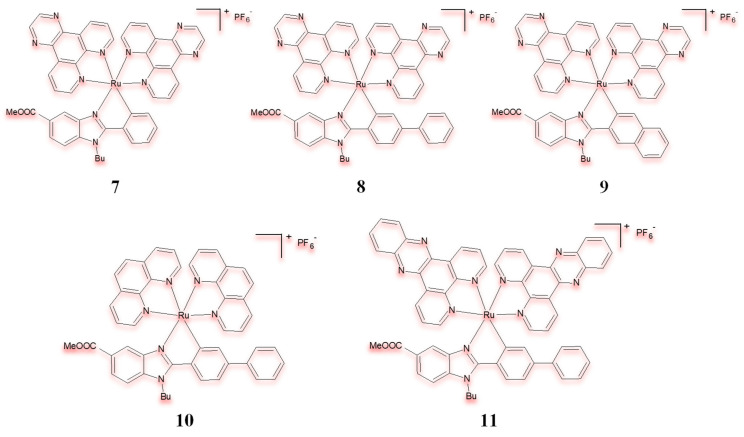
Schematic of structures of **7**–**11** [89].

To address the above problems, a team led by Ruiz [89] synthesised a group of heteroleptic complexes of cyclometalated Ru(II) with different N,N-donor polypyridyl ligands (**7**–**11**) (Figure 4).

The anticancer activity of the tested Ru(II) compounds was evaluated in vitro against the following human cancer cells: A2780, A2780cis, HeLa, MB-MDA-231 and CHO. Data analysis revealed that tested compounds possessed very good cytotoxicity against the selected cancer cells (IC_50_ values 9–230 μM) [89]. The above compounds have been investigated as biological photosensitisers of green light. Biological studies have shown that complexes **8** and **10** exhibit the highest activity and excellent phototherapeutic indices. The studies presented here suggest that heteroleptic cyclometalated Ru(II) compounds with large N,N-donor polypyridyl ligands can be good photosensitisers for tumour suppression under hypoxic conditions using low-energy green light. Moreover, in normoxic conditions, H_2_O_2_ is the major compound photogenerated by ruthenium complexes, along with OH^‧^ and ^1^O_2_.

**Figure 5:**
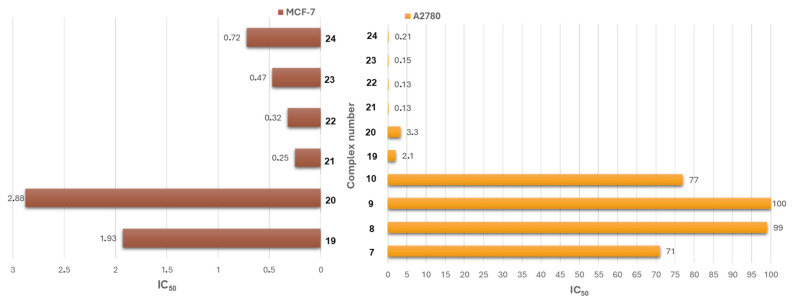
Visual representation of cytotoxic activity of Ru(II) complexes with selected ligands against A2780 and MCF-7 cells (IC_50_).

In order to illustrate the influence of similar ligands on the cytotoxic properties of Ru(II) complexes, IC_50_ values were compared for the following cancer cell lines: A2780 and MCF-7 (Figure 5).

**Scheme 2:**
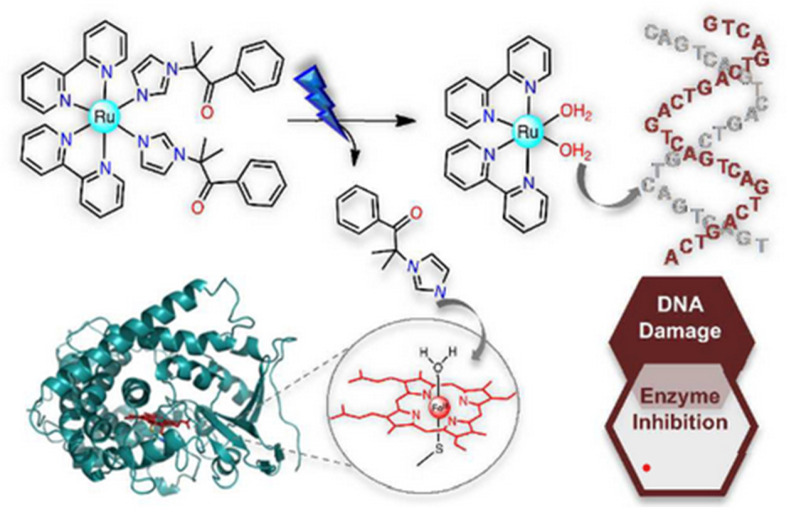
Design of dual action inhibitors [91] (RSC permission).

Ru(II) complexes are another group of prodrugs with interesting mechanisms of action that are used as PSs in the photoactivated chemotherapy (PACT) method. This method differs from PDT because it does not require the presence of molecular oxygen, which is essential in PDT. Namely, in this therapy, according to Scheme 2, only PS and light are used, and the biological activity of these types of Ru(II) complexes results from the process of dissociation of labile ligands under the influence of light, resulting in the formation of a released ligand and the remaining part of the complex. The ruthemium(II) complex presents dual action; namely, the released ligand can inhibit enzymes, including the P450 enzyme, while the remainder of the compound binds directly to DNA through the Ru(II) ion. Notably, the ruthenium complex released from the ligand in the biological environment can be coordinated by solvent molecules (Scheme 2) [4,10,90,91].

**Figure 6:**
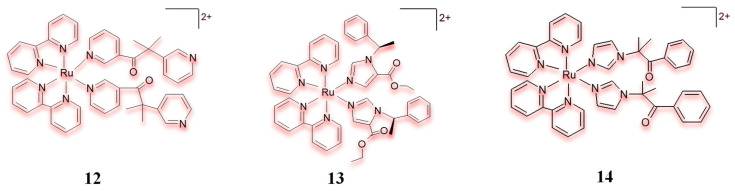
Schematic of structures of the complexes **12**–**14** [91].

Examples of compounds that can be used in the PACT method include the Ru(II) complexes (Figure 6) obtained by Zamora and co-authors [91].

In their paper [91], the authors showed that the inhibition of cytochrome P450s significantly affects the apoptosis of cancer cells. These compounds (Figure 6), when activated by light, have a dual mechanism of action that is consistent with Scheme 2, described above. The released ligand acts as an inhibitor of cytochrome P450s, and the metal centre coordinates directly to DNA [91]. The authors also showed that a compound containing imidazole-substituted ketone groups in the ligand had the best properties for inhibiting protein synthesis. Studies on the design of prodrugs for PACT therapy based on model Ru(II) complexes have shown that it is important to select ligands that function as inhibiting agents for P450 enzymes. This is because interactions between the released ligand and the enzyme can synergize with DNA-damaging agents in cancer cells, thus overcoming the problem of drug resistance. To date, P450 inhibitors have been used in medicine as inhibitors in the treatment of breast and prostate cancer, and Cushing’s disease [92,93,94]. Unfortunately, P450 inhibitors are not selective and can remain in the body for a long time, which may cause undesirable drug interactions and hormonal changes [95]. Therefore, the Ru(II) complexes discussed here as dual-acting prodrugs may provide a novel therapeutic solution for inhibiting P450-type enzymes while inducing DNA damage in cancer cells.

### 2.3. Half-Sandwich Ru(II) Complexes and the Influence of Organic Ligand Modification on Cytotoxicity

**Figure 7:**
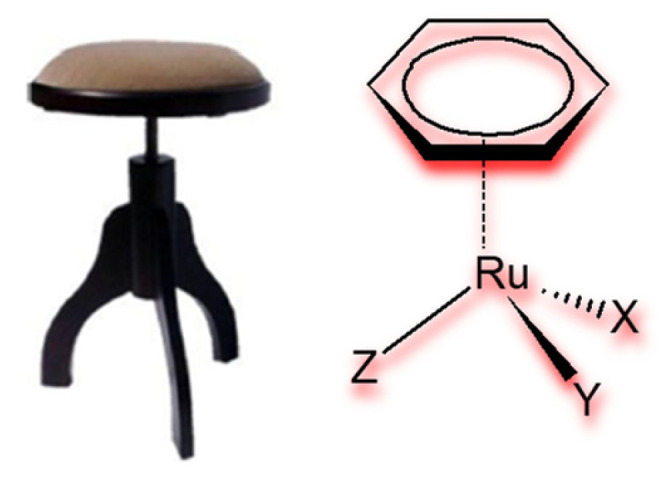
Model structure of the arene half-sandwich Ru(II) complex [96].

The organometallic arene Ru(II) complexes are prospective group of complexes with the pseudo-octahedral geometry of the so-called piano stool. The appropriate choice of the arene substituents and mono- and bidentate ligands in half-sandwich compounds of the [(η^6^-arene)Ru(XY)(Z)]^n+^ type (Figure 7) allow the precise modulation of the physicochemical and biological properties of the compounds [96,97,98,99,100].

**Figure 8:**
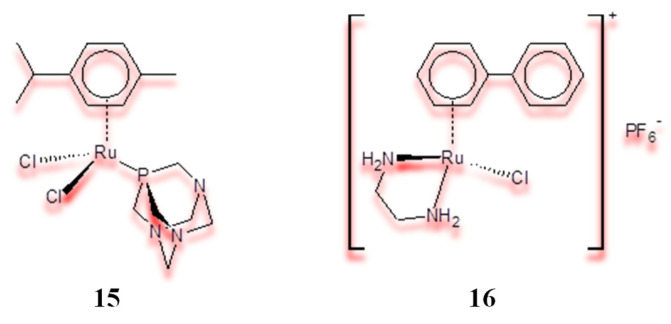
Schematic of structures of arene Ru(II) complexes RAPTA-C (**15**) and RAED (**16**) [46].

Analysis of the model structure in Figure 7 suggests that complexes of this type contain the following: the arene ligand, which provides the lipophilic character of the compound; the monofunctional ligand Z, which provides the activation site; and the bifunctional ligand XY, or the monofunctional ligands X and Y, which largely modulate the biological properties of the compound [42,43,96]. Recent reports suggest that ruthenium(II) arene complexes may exhibit a different type of anticancer MoA, as their biological action is likely to be multitargeted. Indeed, half-sandwich ruthenium(II) complexes may be activated by the substitution of a monodentate Z ligand (e.g., Z = Cl^−^) with water molecules, which make possible the binding of the complex to DNA or other biological molecules [42,92]. In addition, the biological activity of half-sandwich compounds may also be related to interactions with different biomolecules [101,102,103,104]. Consequently, organometallic ruthenium(II) half-sandwich complexes represent group of potential chemotherapeutics and, owing to the highly lipophilic and labile nature of their ligands, may represent an important alternative to the platinum anticancer drugs used [97,105,106,107]. They possess unique physicochemical and biological properties, research on which was initiated by the Dyson and Sadler teams [108,109,110]. Scientists have synthesised two new Ru(II) complexes, RAPTA-C and RAED, which are currently in preclinical testing (Figure 8) [111,112,113].

**Scheme 3:**
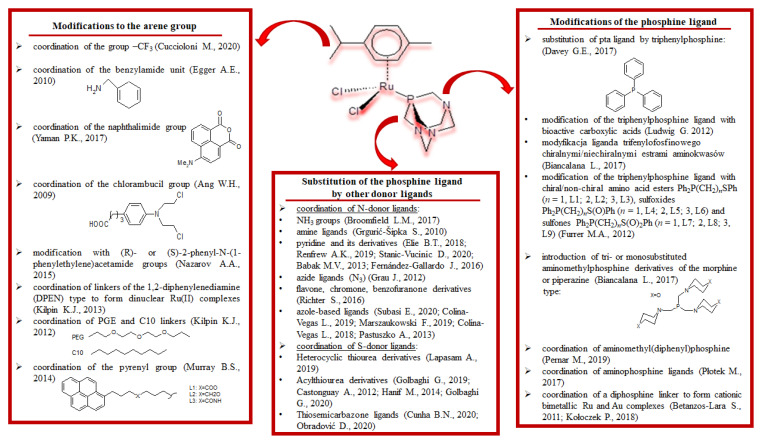
Model of complex RAPTA C and its studied modifications [120,121,122,123,124,125,126,127,128,129,130,131,132,133,134,135,136,137,138,139,140,141,142,143,144,145,146,147,148,149,150,151,152,153,154,155,156,157].

The first compound studied by Dyson’s group shows potent anticancer properties to prevent cancer metastasis in the body. In contrast, the RAED compound (Sadler’s group) inhibits primary tumour growth [114,115]. The first phase of interacting with DNA involves the aquation of metal–halogen bonds. Moreover, the RAED compound is more likely to bind to DNA, but the RAPTA is more likely to target the nucleosome [116]. The RAPTA complex is also thought to have greater reactivity than the RAED because of the two labile halogen ligands in its structure. In addition, further studies have shown the low toxicity of the complexes to healthy cells, as evidenced by their ability to effectively remove the tested compounds from the bloodstream [117,118]. Given the high activity level of the RAPTA-C compound in inhibiting cancer metastasis, researchers have focused on piano-stool complexes with other ligands based on phosphorus as donor atoms to Ru(II). The modifications shown in Scheme 3, among others, and changes in the model complex, were found to modify the biological properties of the resulting combinations. Therefore, an attempt was made to determine (i) the influence of additional substituents in the ring of the arene ligand, (ii) the replacement of the PTA ligand by other phosphorus ligands and (iii) the effect of the modifications of the PTA ligand in the model compound.

Notably, the results obtained using RAPTA analogues [119,120,121,122,123,124,125,126,127,128,129,130,131,132,133,134,135,136,137,138,139,140,141,142,143,144,145,146,147,148,149,150,151,152,153,154,155,156] allowed correlations to be identified between the structure of the arene and the ligands included in the complexes, and the activity of the chemotherapeutic agent tested (IC_50_). The main factors that influenced the cytotoxicity were steric factors and changes in electron density caused by the type of arene ring substituent [130,131,151,152,153,154,155,156]. Predominantly electronegative substituents, which decrease the electron density of the arene ring, were found to increase the cytotoxicity of complex. In addition, Ru(II) complexes with the ligands modified with phosphorus donor atom [126,127,128,129,145,147,148,149,150] substituents in the form of different alkyl chain lengths did not alter the cytotoxicity. On the other hand, phosphorus ligands containing aromatic rings and -CH_2_OH groups did affect the physicochemical properties of Ru(II) complexes. Compared with RAPTA, the complexes underwent only minor hydrolysis, while they showed good catalytic properties in the reaction with NADH. Among others, complexes containing phosphines with -OCH_3_ groups in the phenyl rings showed increased catalytic activity in the oxidation process of NADH to NAD^+^.

### 2.4. Ruthenium(II) Complexes Act on More than One Biological Target (Multiple Targets)

**Figure 9:**
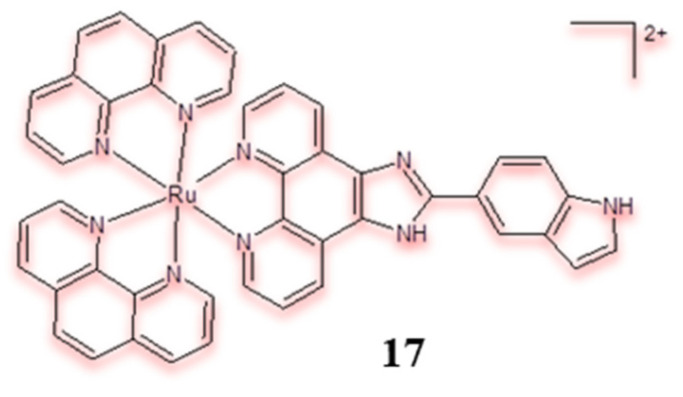
Polypyridine complex of **17** [158].

As mentioned earlier, compounds that act on more than one biological target (multiple targets) to overcome cellular resistance mechanisms are considered more effective therapeutics [157,158,159]. Recent discoveries in ruthenium-based chemotherapeutics research with a complex MoA have led researchers to develop new polypyridine and cyclometalated ruthenium(II) complexes. Liu and coworkers obtained a new Ru(II) compound (**17**) (Figure 9) , which exhibited a dual mechanism of anticancer action, (1) through apoptosis via mitochondrial pathways and (2) through interaction with DNA involving blocking telomeric DNA in the G-quadruplex conformation. The complex showed anticancer activity towards the HeLa cancer cell line (IC_50_ = 11.7 ± 1.2 μM) and cisplatin (7.6 ± 0.4 μM). Moreover, the compound showed less activity than cisplatin towards the healthy mouse embryonic fibroblasts.

**Figure 10:**
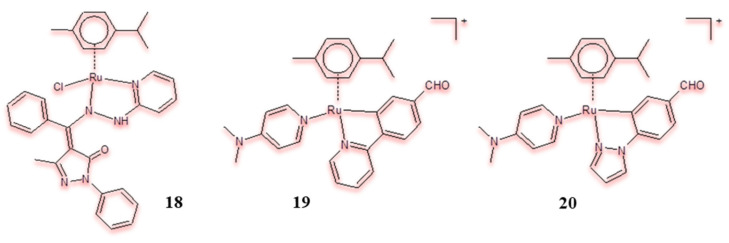
Organometallic Ru(II) complex analysed by Eleuteria’s team (**18**) [157] and Ru(II) complexes with C,N-donor ligands (**19**, **20**) [160].

Furthermore, more advanced methods have shown that this Ru(II) complex efficiently penetrates HeLa cells, first accumulating in lysosomes and then localising to more sensitive organelles, such as the nucleus. The complex interacts with DNA and inhibits the activity of the telomerase enzyme, which inhibits the stages of DNA gene expression [158]. M. Eleuteria’s team has obtained organometallic Ru(II) arene complexes, such as **18** [157] (Figure 10), which show interesting multitarget activities that can overcome cellular resistance mechanisms.

The studied complex **18** has been shown to bind reversibly to albumin and other intracellular molecular targets, such as cholesterol enzymes (HMGR), the proteasome and DNA. Studies on the in vitro cytotoxic activity of the Ru(II) complex towards cancer cell lines (MCF-7, MCF-10A and MCF7-CR) revealed that the complex showed the highest cytotoxic activity towards MCF-7. In addition, the compound showed activity against the cisplatin-resistant MCF-7CR cell, with much lower activity against the noncancerous MCF-10A cell. The cytotoxic MoA of the tested compound was tentatively determined to involve the partial induction of cell cycle progression arrest in the G0/G1 phase, resulting in the accumulation of the compound in MCF-7 and MCF-7CR cells [157].

**Figure 11:**
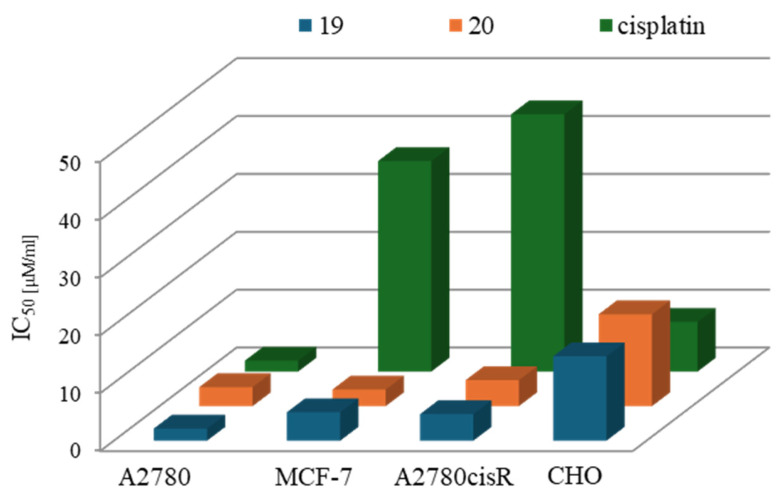
IC_50_ values [μM] for **19**, **20** and cisplatin after 48 h of incubation.

In recent years, researchers have focused their attention on Ru(II) arena complexes with C,N donor ligands. Noteworthy are Ru(II) compounds **19**, **20** [160] (Figure 10), which have been investigated for a new mechanism of action (MoA). The cytotoxicity of these compounds was evaluated against several human cancer cell lines, including A2780, A2780cisR and MCF-7, as well as noncancerous BGM and CHO cells. The compounds showed high in vitro antitumour activity against all the tumour cells tested (see Figure 11). In addition, protein synthesis studies in A2780 cancer cells using flow cytometry and fluorescence intensity measurements revealed translation inhibition, followed by G1/S phase arrest and mitochondrial caspase-dependent apoptosis. These factors likely induce cell death and are the main model for the action of cytostatic drugs.

**Figure 12:**
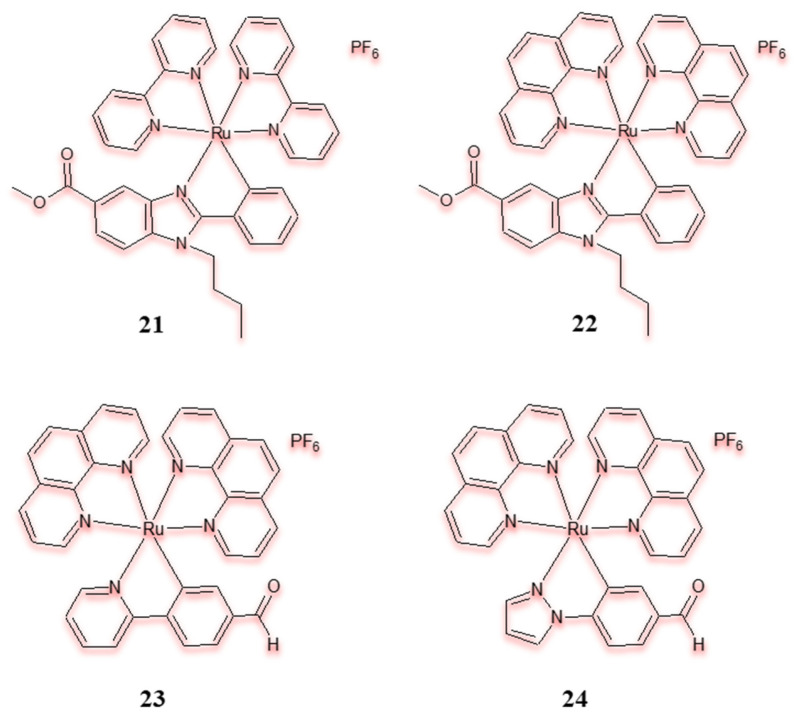
Examples of cyclometalated Ru(II) complexes (**21**–**24**) [161].

Brabec and Ruiz [89,161] presented a group of kinetically inert Ru(II) complexes **21**–**24** (Figure 12), which contain C,N-donor functional ligands. The resulting complexes are highly potent cytotoxic agents against selected human cancer cells.

**Figure 13:**
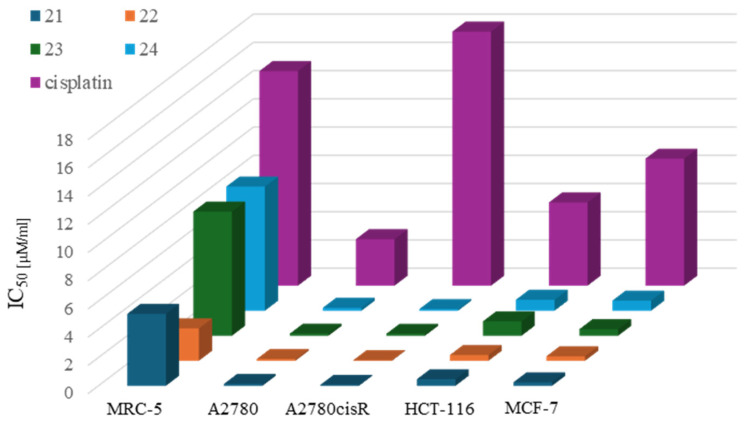
IC_50_ values [μM] for **21**–**24** and cisplatin after 72 h of incubation.

All ruthenium compounds showed significantly higher anticancer activity towards A2780, A2780cisR, HCT-116, MCF-7 and healthy MRC-5 cells compared to towards cisplatin (see Figure 13) [161]. In addition, the RF resistance ratio was 0.35–0.94 for the ruthenium complexes, whereas for cisplatin, the RF resistance ratio was significantly greater, at 5.28. These data suggest that the MoA of the Ru(II) compounds is different from that of cisplatin; thus, the compounds studied can successfully overcome the resistance mechanisms that counteract the effects of cisplatin. In addition, the ruthenium complexes have been shown to cause mitochondrial dysfunction, which is also associated with ROS production. Thus, it is possible that cyclometalated Ru(II) complexes have a dual mechanism of action against cancer cells, primarily through inhibiting proteosynthesis and, to a lesser extent, through disrupting mitochondrial function. To conclude this chapter, we emphasise that the above literature review of research into ruthenium complexes in the search for effective chemotherapeutics illustrates the enormous efforts being made by scientists to combat the modern scourge of cancer.

## 3. Potential Rhodium-Based Chemotherapeutics

Rhodium is not included in the group of metals with established biological functions essential for humans or other organisms. However, research into the biological effects of rhodium complexes began with the study of the interactions between rhodium compounds and casein proteins in 1958 [162], and, shortly thereafter (1974–1977), rhodium(II) complexes were first investigated as potential anticancer drugs or enzyme inhibitors [163,164]. The literature indicates that current research is concerned mainly with (i) Rh(III) complexes in which the metal has a structural function in (a) determining the inhibition of essential enzymes involved in tumour cell processes, (b) optimising the geometrical conditions for Rh(III)-DNA complex interactions, or (c) influencing the catalytic activity of NAD^+^/NADH reduction; (ii) dimeric Rh(II) complexes, whose the key biological role results from the structure of the compound and types of ligands; and (iii) polynuclear ligand-bridged rhodium complexes.

### 3.1. Rh(III) Complexes in Which the Metal Has a Structural Function

**Figure 14:**
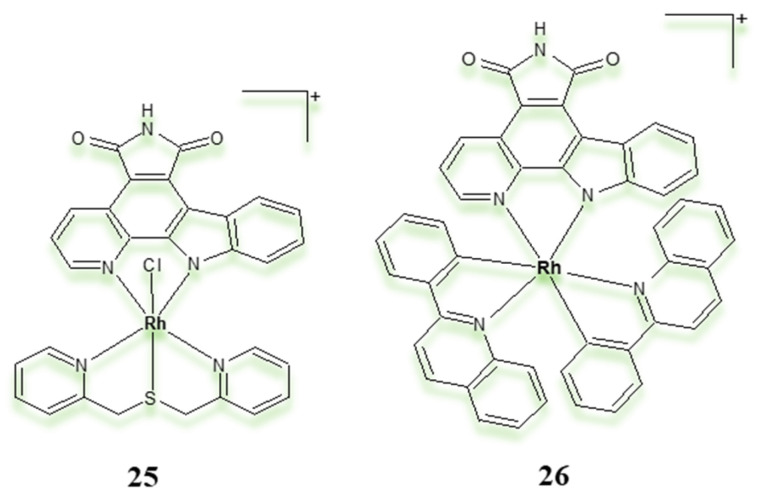
Rhodium(III) complex as an inhibitor of Src kinase (**25**) [24] and as an inhibitor of the enzyme NEDD8 (**26**) [162].

Meggers [24] was the first to recognise that the central ion in Rh(III) complexes has a structural role in interactions with biological systems. He reported that optimal geometric conditions for Rh(III) complex–DNA interactions is possessed through the octahedral structure of Rh(III) complexes. As a result of this structural optimisation, the rhodium complex (**25**) with staurosporine was found to have the ability to inhibit the protein tyrosine kinase Src (Figure 14).

A similar structural approach was used to develop the rhodium inhibitors (**26**) of the enzyme NEDD8 (the enzyme that controls ubiquitin ligase activity), which inhibited inflammation in vivo in a mouse model (Figure 14).

**Figure 15:**
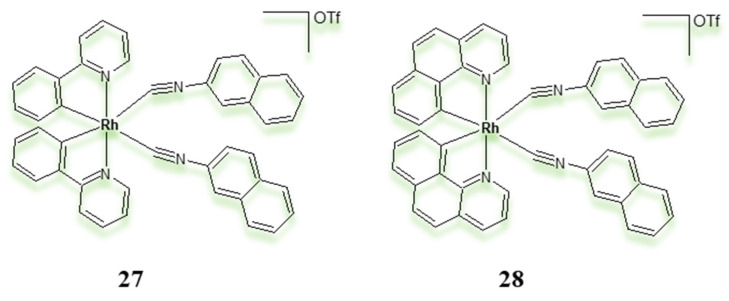
Cyclometalated Rh(III) complexes as an inhibitors of kinase (JAK2) [165].

Rh(III) cyclometalated complexes are also being intensively studied as kinase inhibitors (JAK2) (Figure 15) [165].

The above-cited rhodium complexes were found to be strong and selective inhibitors of enzyme activity (see also for details review [166]).

**Figure 16:**
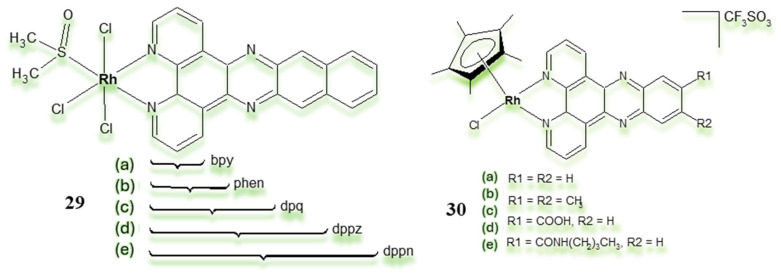
Schematic of structures of (**29a**–**e**) [171] and polypyridyl half-sandwich rhodium(III) complexes (**30a**–**e**) [175].

Scientific reports [167] suggest that rhodium complexes containing polyaromatic ligands exhibit high levels of activity towards a range of cancer cells. The polyaromatic ligands allow for intercalation into DNA, increasing the log *p* values of the compound, and can affect the redox properties of metal complexes. With this in mind, Sheldrick et al. [168,169,170,171] analysed anticancer properties of series of Rh(III) polypyridyl complexes (Figure 16).

Complexes with the general formula mer-[RhCl_3_(DMSO)(pp)] showed strong cytotoxicity against MCF-7 and HT-29 cancer cells. This activity increased with the number of aromatic rings: bpy < phen and dpq < dppz < dppn. In addition, further studies on these compounds revealed their accumulation in mitochondria, suggesting possible oxidative damage to mitochondrial DNA [171,172]. To further investigate the effect of polypyridine ligand size, Sheldrick and colleagues synthesised a series of half-sandwich rhodium(III) polypyridyl complexes (Figure 16) [168,173,174].

**Figure 17:**
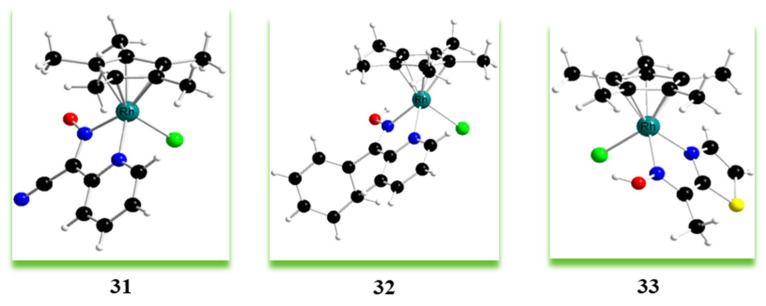
Molecular structures of **31** [CCDC 148625], **32** [CCDC 1486256] and **33** [CCDC 1486258] [177].

Research on piano-stool complexes was made possible by the use of a pentamethylcyclopentadienyl ligand (Cp* = η^5^-C_5_Me_5_) in their construction, which stabilises the compound, increases its lability and provides the hydrophobic part of the complex. Biological experiments with the above complexes confirmed that in vitro cytotoxicity is connected mainly with the polypyridyl ligand. The methyl substitution of the dppz ligands in [(η^5^-C_5_Me_5_)RhCl(dppz)]^+^ resulted in a significant increase in cytotoxic activity. Compared with the complex **30a**, the complex **30b** presented an increase in cytotoxic activity against the MCF-7 and HT-29 cell lines, respectively [176]. Recently, Kollipara and coworkers [177], using the chelating ligands 2-pyridylphenoxime, 2-pyridylphenoxime and 2-thiazolylmethyloxime, obtained half-sandwich Rh(III) complexes **31**–**33** (Figure 17). The resulting complexes were characterised analytically, spectroscopically and structurally.

**Figure 18:**
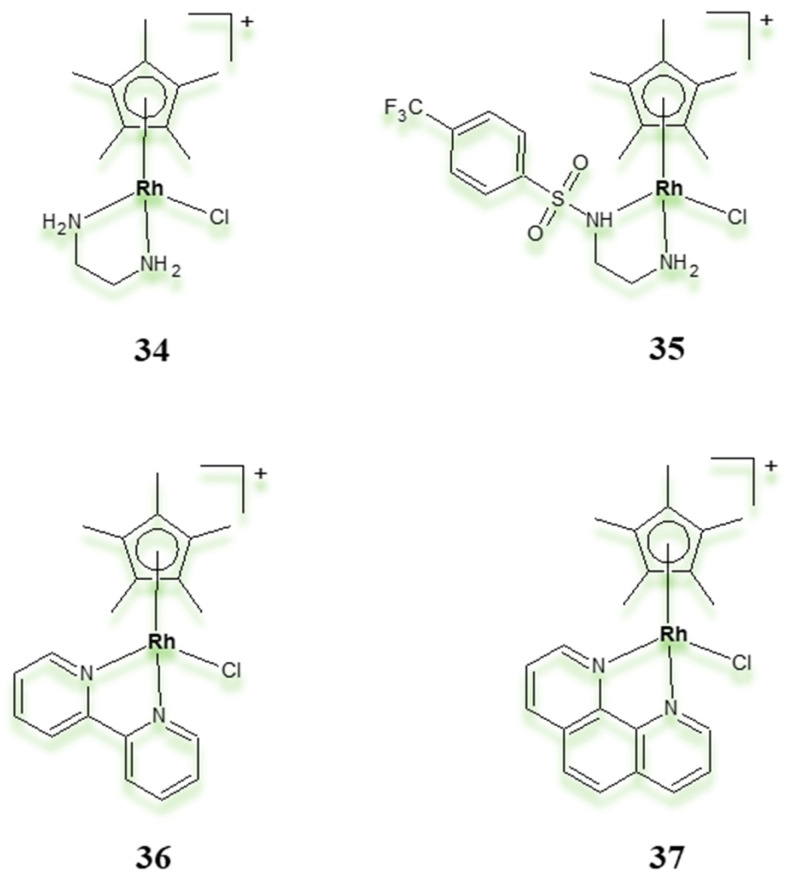
Rh(III) complexes as biocatalysts [178].

In vitro analyses of complexes towards HT-29 and MIAPaCa-2 revealed that cytotoxicity depends on the type of chelating ligand. The activity decreased in the order {pyC(CN)NOH} > {pyC(Ph)NOH} > {tzC(Me)NOH}. Complex **31** was the most active (HT-29, IC_50_ = 23.74 ± 4.25 μM; MIAPaCa-2, IC_50_ = 9.16 ± 2.89 μM). The research of Sadler’s team [178] on the use of Rh(III) compounds as biocatalysts for the reduction in NAD^+^ to NADH using formate as a hydrogen anion source (ligand hydride) under biological conditions is noteworthy. The researchers studied the piano-stool complexes shown in Figure 18.

**Figure 19:**
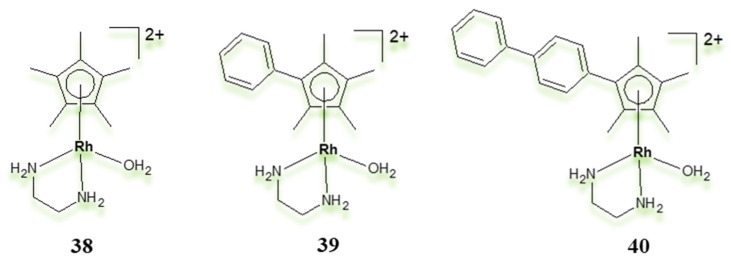
Rh(III) complexes containing ethylenediamine (en) [178].

Interestingly, the bpy-containing complex (**36**) exhibited the greatest catalytic activity (37.4 ± 2 h^−1^). Furthermore, it was found that complexes containing ethylenediamine increased these anticancer properties when additional arene substituents were introduced into the Cp^X^ ring (Figure 19).

At the same time, it should be mentioned that a more detailed review summarising the field of research on potential chemotherapeutic agents with half-sandwich Rh(III) compounds can be found by interested readers in the review paper by P. Štarh and co-authors [12].

### 3.2. Dimeric Rh(II) Complexes with Metal–Metal Bonds

**Figure 20:**
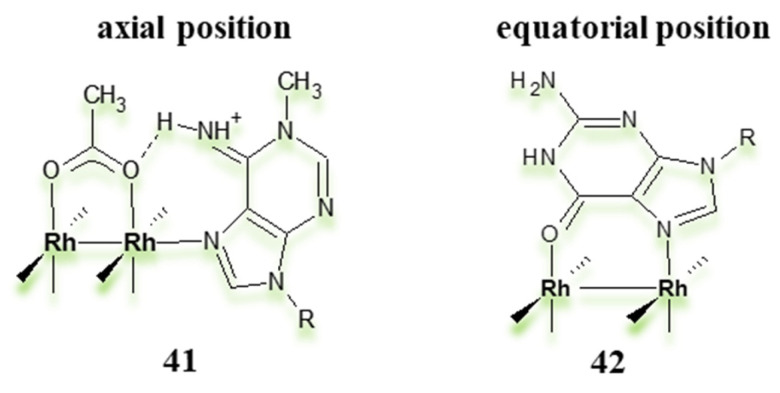
Direct interaction with DNA bases at **41** axial and **42** equatorial sites of paddle-wheel complexes [162].

In addition to octahedral Rh(III) complexes, dimeric Rh(II) complexes that target DNA constitute another important area of research in bioorganic chemistry. These compounds are characterised by a classical paddle-wheel structure [179]. The biological properties of Rh(II) dimers are due, in part, to their molecular structure, which allows them to interact with the bases of the DNA nucleic chain (Figure 20).

**Figure 21:**
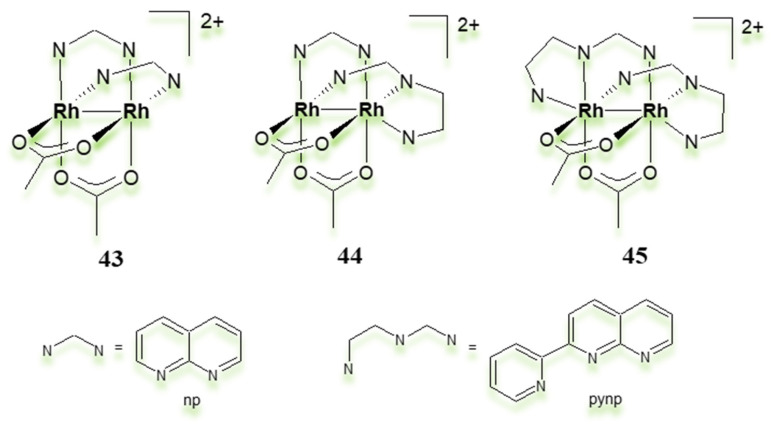
Rh(III) paddle-wheel complexes **43**–**45** [176].

Direct interaction with DNA bases occurs at the axial sites of the complexes, where ligand exchange takes place (Figure 20) [162,180]. In some cases, the substitution of the DNA base can also occur at the equatorial position. This occurs in complexes containing labile ligands in the equatorial position, where the DNA base initially binds in the axial position, and the axial ligand is subsequently converted to the equatorial position [181,182]. Recent studies have shown that paddle-wheel complexes with the ligand-mediated blocking of labile sites in the complex lack biological activity [176]. The main goal of the study was to compare the DNA interactions of complexes with one or two available coordination sites at axial position **43** and **44** with that of a complex in which the axial sites are blocked, **45** (Figure 21).

Studies have shown that complex **43** binds most strongly to DNA (K_b_ = 5.6 × 10^5^ M^−1^), and, thus, the ability of the complexes to interact with DNA and inhibit transcription in vitro has been shown to depend on the availability of free axial sites where biologically active Rh(II) dimers can interact directly with DNA bases.

**Figure 22:**
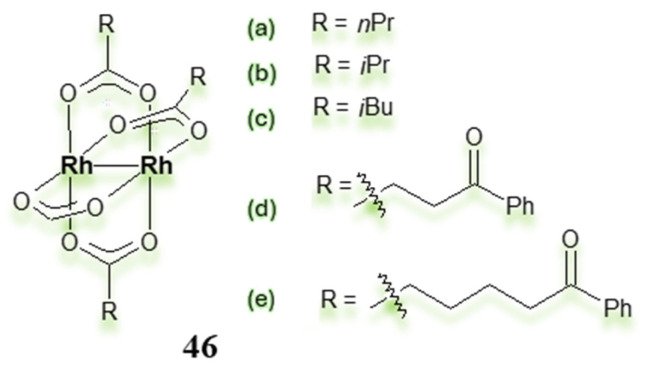
Dimeric Rh(II) complexes as UPS inhibitors [8].

Recently, Chan and coworkers [8] presented a series of rhodium(II) dimeric complexes (**46a**–**46e**) (Figure 22) as inhibitors of the UPS (ubiquitin–proteasome system) [8,183,184].

**Figure 23:**
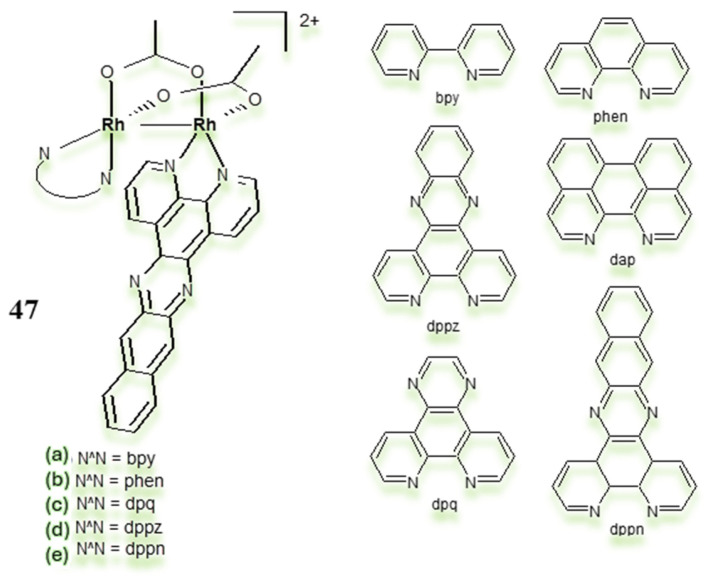
Rh(II) paddle-wheel complexes with N,N-donor ligands **47a**–**e** [175].

The inhibitory capacity of the complexes (Figure 22) against the UPS was strongly related to the cytotoxicity of the compounds. Interestingly, the replacement of carboxylates acting as bridging ligands by carboxamides abolished the inhibitory effect on the UPS, suggesting that the auxiliary ligand (carboxamides) plays an important role in the inhibition capacity of such complexes. Dunbar and coworkers [175] conducted extensive biological studies on heteroleptic rhodium(II) dimeric complexes (Figure 23).

**Figure 24:**
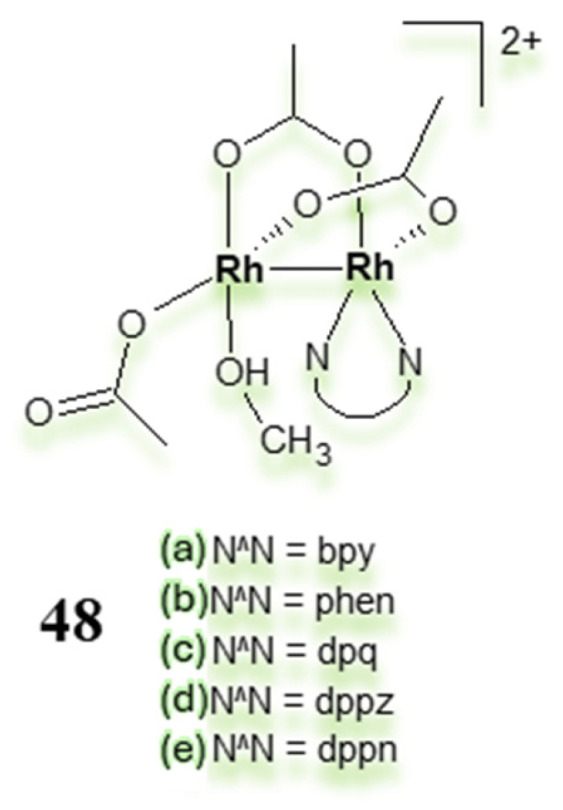
The schematic structures of Rh(II) dimers [185].

Some of the compounds studied (Figure 23) intercalated with the DNA, while the other complexes interacted with the DNA via electrostatic interactions. Additionally, complexes **47d** and **47e** exhibited significantly lower toxicity in the dark towards Hs-27 cell (**47d** LC_50_ = 355 μM and **47e** LC_50_ = 384 μM). The cytotoxic activity of the **47d** and **47e** against Hs-27 after irradiation with visible light significantly increased compared to in darkness (21- and 24-fold for **47d** and **47e**, respectively). The significant increase in the cytotoxicity of the studied complexes after irradiation makes them potentially useful in PDT. Similar studies were carried out by Dunbar’s team [185], who synthesised six Rh(II) dimers (Figure 24).

Complexes **48d** and **48e** were shown to intercalate with DNA bases as well as to bind covalently to DNA, and could, therefore, be considered a new type of potential chemotherapeutic agent. The greater strength of the interaction with DNA, the more cytotoxic the complex. The exception among the tested complexes was compound **48e**, which differed from the others by having a positive log *p* value (log *p* = +0.91 ± 0.01) and by interacting the most strongly with DNA (binding constant K_b_ = 9.7‧10^5^ M^−1^). However, it was not the most cytotoxic towards the cancer cells tested, probably owing to its intracellular localisation.

**Figure 25:**
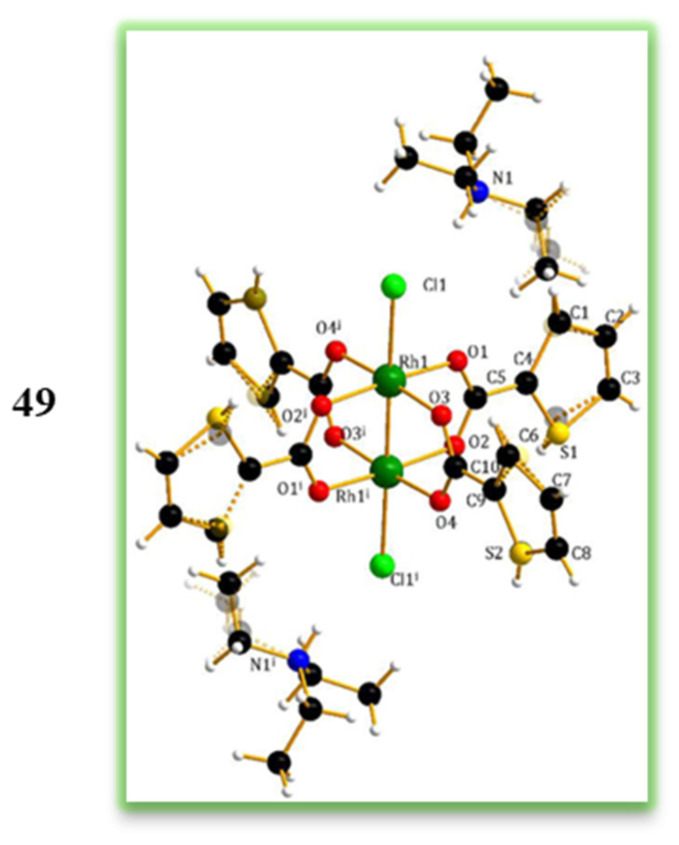
Molecular structures of **49** [CCDC 1844129] [186].

Notably, Masternak and co-authors [186] presented a new simple method for the synthesis of paddle-wheel-type rhodium(II) complexes with a deprotonated form of thiophene-2-carboxylic acid. The authors used metallic rhodium, which acts as both a catalyst and reductant of Rh^3+^ to Rh^2+^ ions, in the self-assembly method of synthesis to obtain an anionic dimer (Figure 25).

The obtained complex exhibits cytotoxicity towards all tested cancer cells and appears to be a promising chemotherapeutic agent for the treatment of MV-4-11 (IC_50_ = 4.02 μM). Furthermore, spectroscopic studies revealed that **49** can interact non-covalently with DNA by binding in the Rh(II)-CT-DNA dimer groove. According to the literature [187], this site is preferred for linear structures, and, in the case of the rhodium dimer, linearity is observed due to the geometry of the Cl(1)-Rh (1)-Rh(1i)-Cl(1i) (179.999(18)°) unit, which, according to structural studies, retains its linearity.

Recent reports in the literature suggest that dimeric rhodium(II) complexes may find applications as potential photosensitisers in antitumour PDT. It has been reported that the ^1^O_2_ generated by Rh complexes causes the death of photoactive tissue. With this in mind, Dunbar and coworkers [175] further investigated the photophysical activities of previously obtained Rh(II) complexes with polypyridyl ligands (see Figure 23) [175]. The complexes studied were found to produce ROS and to damage DNA upon irradiation. The toxicity of **47a**–**e** to Hs-27 increases after exposure to visible light. This effect is particularly pronounced for complexes **47d** and **47e**, whose toxicity increases by 21- and 24-fold, respectively. Thus, the properties of these complexes make them potential sensitisers in PDT.

**Figure 26:**
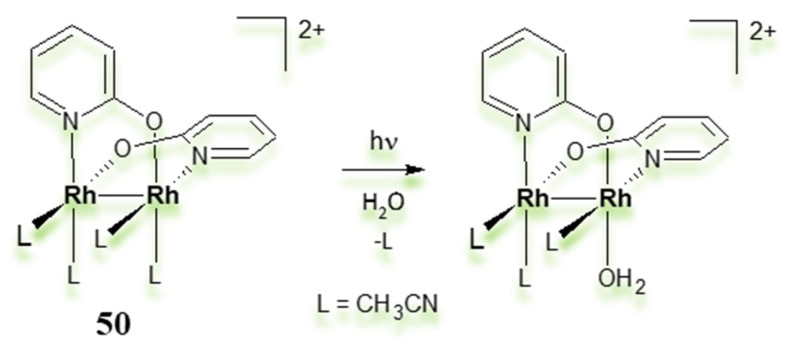
Rh(II) dimer with oxopyridine (**50**) [188,189].

C. Turro and colleagues studied Rh(II) dimer with oxopyridine **50** (Figure 26) and reported that photoirradiation facilitates a ligand exchange process that results in interactions of the complex with DNA [188,189]. The complex has a short (<1 ns) excited state lifetime, which prevents the generation of ^1^O_2_, and exhibits an oxygen-independent cell-killing effect, mainly through photoinduced ligand exchange, which is important for cancer control under hypoxia [190].

### 3.3. Polynuclear Ligand-Bridged Rhodium Complexes

**Figure 27:**
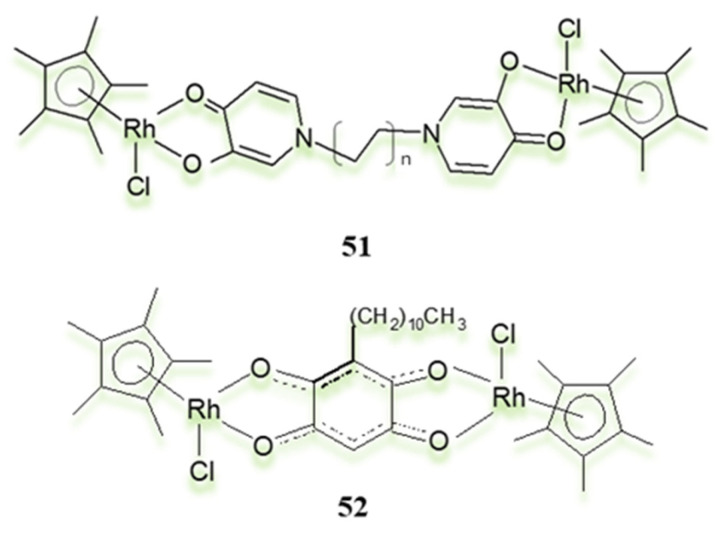
Dinuclear rhodium complexes **51** (*n* = 4, 6, 8, 12) [193] and **52** [197].

The inspiration for research into multinuclear complexes containing platinum group metals, particularly rhodium, was the trinuclear platinum complex BBR3464, which was selected for preclinical studies [191,192]. It was concluded that the trinuclear Pt(II) complex, which has less toxic effects on healthy cells, could become an effective chemotherapeutic agent for the therapy of cancers resistant to the presently used classical platinum compounds. Inspired by the above studies, C. G. Hartinger and his team [193] obtained and characterised a group of binuclear complexes with bridging ligands with intermetallic bridges of different lengths (*n* = 4, 6, 8, 12) to modify the lipophilicity. The dinuclear complex with bridging ligand *n* = 12 (Figure 27) [193] exhibited significantly higher cytotoxic activity (HCT-116, IC_50_ = 0.20 ± 0.02 μM; NCI-H460, IC_50_ = 0.05 ± 0.01 μM; SiHa 0.46 ± 0.03 μM; SW480, IC_50_ = 0.26 ± 0.07 μM) than its analogous complexes described in [193]. Furthermore, in advanced in vitro studies, the compound generated ROS and caused DNA damage at low concentrations but showed low toxicity compared to cisplatin. Similar (Figure 27) high-cytotoxicity properties ((IC_50_ = 0.7 μM (B16F10, A549); IC_50_ = 0.8 μM (MCF-7)) against selected cancer cell lines were exhibited by the dinuclear complex **52** which was used as a precursor to obtain three types of metallacages rhodium: tetranucleophiles [194,195], hexanucleophiles [196,197] and octanuclear metallacubes [198].

**Figure 28:**
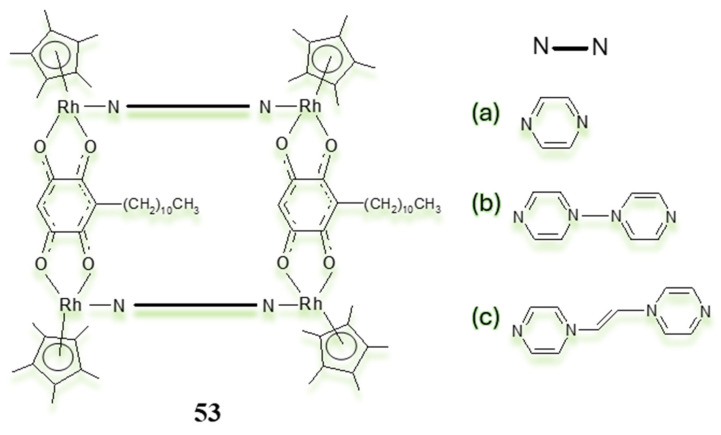
Tetramer rhodium complexes [195].

The resulting four-nucleated rhodium complexes **53a**–**c** (Figure 28) were evaluated via in vitro assays against selected DU-145, A-549, HeLa and normal HEK-293 cells.

All the rhodium complexes tested showed superior cytotoxic activity and very good selectivity between cancerous and healthy cell lines. The most active rhodium complex was **53a** (DU-145 IC_50_ = 0.54 ± 0.2 µM; A-549 IC_50_ = 0.50 ± 0.1 µM; HeLa IC_50_ = 0.52 ± 0.2 µM) and for the normal cell line HEK-293 (IC_50_ = 62 ± 0.5 µM), the selectivity reached two orders of magnitude. Due to the presence of lipophilic chains in their (**53a**–**c**) structure and the positive charge of the metal, tetramers most likely interact with DNA and the outer mitochondrial membrane.

**Figure 29:**
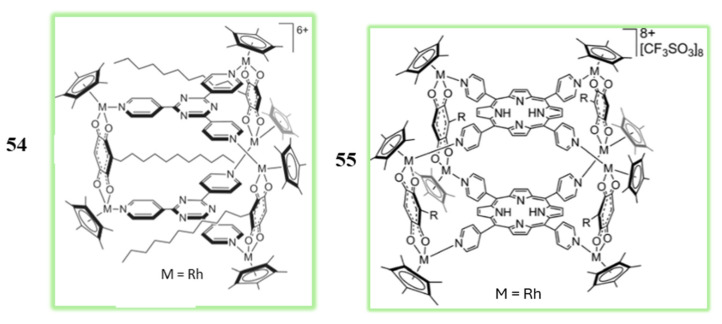
Structural formula of complex **54** [197] with permission of the Wiley-VCH and **55** [198] (RSC permission).

Hexanuclear complexes, which are currently undergoing preclinical trials, deserve special attention. The results of tests on hexanuclear pentamethylcyclopentadienylrhodium(III) **54** (Figure 29) [197] suggest that rhodium(III) metalloprisms have greater antitumour potential (IC_50_ = 0.5 ± 0.3 µM) than their Ir(III) analogues (IC_50_ = 0.7 ± 0.4 µM), causing the efficient induction of cancer cell apoptosis.

In addition, the complex exhibited greater cytotoxic properties in tumour cell lines in contrast to normal cells. In response to the complex’s tumour-reducing effect in mice (C57L6/J), biological studies suggest that this compound has a number of properties necessary to become an anticancer drug [197].

Similarly, the in vitro antiproliferative activity of octanuclear pentamethylcyclopentadienylrhodium metalla-assemblies was evaluated against cancer (MCF-7, B16 and A549) and normal (NIH 3T3) cells. The IC_50_ values obtained for the rhodium complex tested were very low, approximately 0.1 μM. Strong interaction with ctDNA induces apoptosis in cancer cells. This effect results in the high cytotoxicity of the rhodium complex (Figure 29) [198] compared to Ir and Ru analogues.

The above-mentioned studies suggest that large coordination complexes with multiple metal centres display potentially good anticancer properties and warrant further investigation, especially considering their mechanism of action.

**Figure 30:**
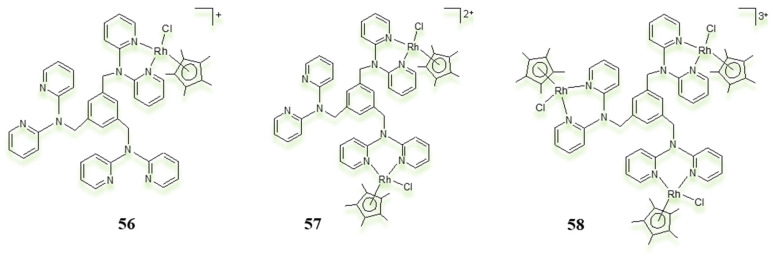
Structural formulas of mononuclear, dimer and trimer rhodium complexes [199].

The research group of B. P. Rao, M. R. Kollipara et al. [199] obtained, characterised and evaluated the biological properties of mononuclear, dimer and trimer rhodium complexes **56**–**58** (Figure 30).

The tested rhodium dinuclear and trinuclear complexes (**57** and **58**, respectively) showed significant anticancer activity towards the tumour cells B16F10 and THP-1. The IC_50_ values of Rh complexes are in low micro molar range compared to their iridium analogues [199]. At the same time, the authors suggest that comparative studies on the MoA of multinuclear complexes Rh-Cp* and Ir-Cp* should be continued.

G. Gupta and co-authors [200] obtained thiolato-bridged dimers (Figure S1).

The tested thiolato complexes **59a** and **59b** showed high cytotoxicity against A2780 (IC_50_ = 1.8 ± 0.2 µM, IC_50_ = 1.1 ± 0.1 µM, respectively) and A2780cisR (IC_50_ = 1.5 ± 0.1 µM, IC_50_ = 1.2 ± 0.1 µM, respectively). The in vitro results obtained indicated the low selectivity of the tested complexes. Similar bioassay results have been obtained for other rhodium thiolato complexes [201,202].

## 4. Research into the Use of Iridium Complexes in Cancer Therapy

**Figure 31:**
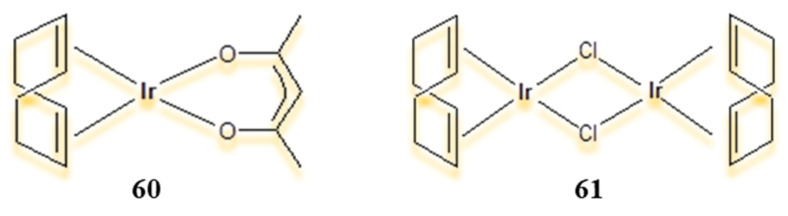
The cyclooctadiene-iridium(I) complexes [204].

In the context of the urgent need to search for new anticancer drugs and research methods for anticancer therapy, iridium(III) complexes have also aroused the interest of researchers [203,204]. Reports in the literature show that the physicochemical properties of iridium allow for the design of effective prodrugs, as an important advantage of these complexes is their relatively good solubility in water, their stability in air and buffer solutions, and relative ease of compound synthesis [205]. A review of the literature shows the following main directions of biological research: (i) half-sandwich Ir(III) complexes as compounds with anticancer properties; (ii) Ir(III) complexes as biocatalysts; (iii) cyclometalated Ir(III) complexes and their use in PDT and PACT therapy; and (iv) cyclometalated Ir(III) complexes as luminescent biological tracers and probes. Notably, research on iridium coordination compounds with anticancer activity was carried out as early as the 1970s. Research focused on flat-square cyclooctadiene complexes of iridium(I) (5d^8^) because of their geometric similarity to cisplatin (Figure 31). The es [Ir(acac)(cod)] (**60**) and [IrCl(cod)]_2_ (**61**) effectively inhibited Ehrlich ascites tumours and lung cancer in mice. The latter has also shown antimetastatic activity in ongoing studies but does not inhibit primary tumours [206,207].

### 4.1. Half-Sandwich Ir(III) Complexes

**Figure 32:**
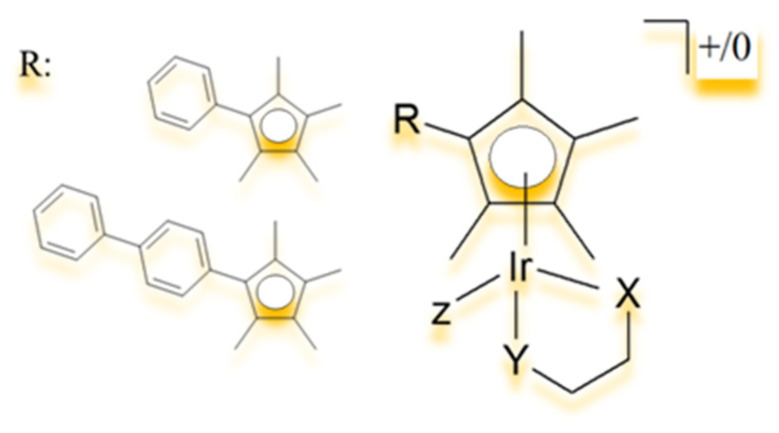
Typical structure of half-sandwich Ir(III) complexes.

Ir(III) half-sandwich complexes include those of the type [(η^5^-Cp*)Ir(XY)Z]^0/n+^ (Figure 32), where Cp* is a pentamethylcyclopentadienyl ligand or derivative occupying three coordination places of the iridium centre and forming hydrophobic part of the complex. Unlike the Ru(II) complexes previously discussed, Ir(III) complexes with benzene derivatives are unstable; therefore, the arene ligand is replaced by a cyclopentadienyl group (Cp*) during synthesis. The presence of the hydrophobic Cp* part of the complex increases its cellular uptake and gives it the ability to interact with the nucleic chain, including intercalation with DNA base rings. Subsequent iridium coordination places (X, Y) are connected with monodonor ligands or N,N-, N,O-, O,O- or C,N-donor chelating ligands. In contrast, the Z ligand is a chloride/halogen anion or an organic donor such as pyridine. The chelating ligand X,Y also stabilises the compound and, in some cases, imparts charge to the complexes. The labile ligand Z, on the other hand, as a leaving group, can undergo an aquatation reaction, allowing the resulting aqua complex to bind more readily to the nucleic acid.

Modifications of mentioned ligands offer great potential for controlling the cytotoxic activity of these complexes [90,204]. In this context, studies on the correlation between the cytotoxicity of a chemotherapeutic agent and the programmed modification of the ligands forming the complexes are needed. Among other topics, the effect of the following factors on the cytotoxicity of the complex has been investigated:

Lengthening the Cp* ring by introducing an additional phenyl or biphenyl substituent [208];

The substitution of N,N-donor by a negatively charged C,N-donor analogue [209];

The replacement of pyridine by a chloride anion (Z) [210].

**Figure 33:**
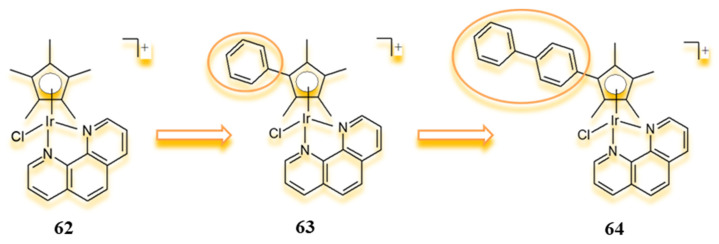
Cyclopentadienyl ring elongation of half-sandwich Ir(III) complexes **62**–**64**.

Studies of the half-sandwich complexes **62**–**64** (Figure 33) confirm that the presence of additional phenyl rings increases the hydrophobicity of the compound and facilitates its passage through cell membranes [208]. In addition, inside the cell, the additional rings can intercalate with the DNA strand. Thus, the half-sandwich complexes Ir(III) **63** and **64** interact with DNA in two ways: they can intercalate with DNA, and they can block replication by directly coordinating with nucleic acid bases, particularly guanine.

**Figure 34:**
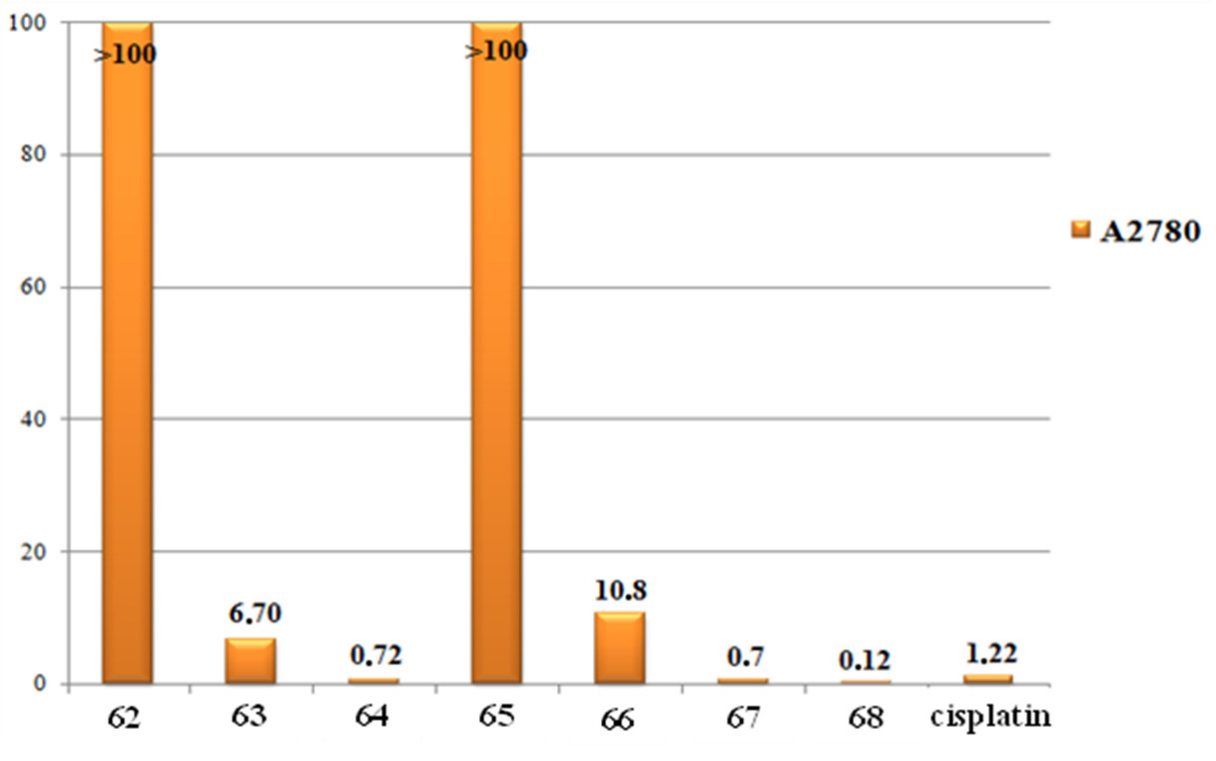
IC_50_ [μM] values for the half-sandwich Ir(III) complexes **62**–**68** and cisplatin. Self-generated graph based on data in [208,209,211].

The data of in vitro cytotoxicity studies on A2780 cancer cell lines (human ovarian cancer) revealed that among the obtained complexes, the compound **64** exhibited the best anticancer properties (Figure 33). Thus, scientific studies have shown that the cytotoxicity of compounds depends on the size of the bound phenyl rings in the complex (Figure 34).

**Figure 35:**
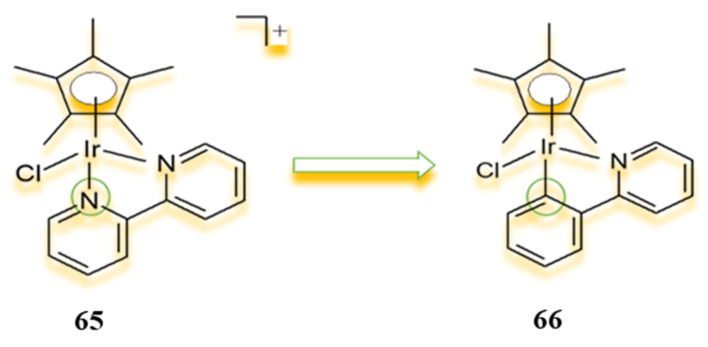
Ligand modifications of piano-stool Ir(III) complexes.

Studies of the effects of the complexes **65** and **66** [209] against A2780 cancer cells have shown that the cytotoxic activity of the compounds can also be increased by substituting the neutral N,N-donor ligand with an anionic C,N-donor (Figure 35).

**Figure 36:**
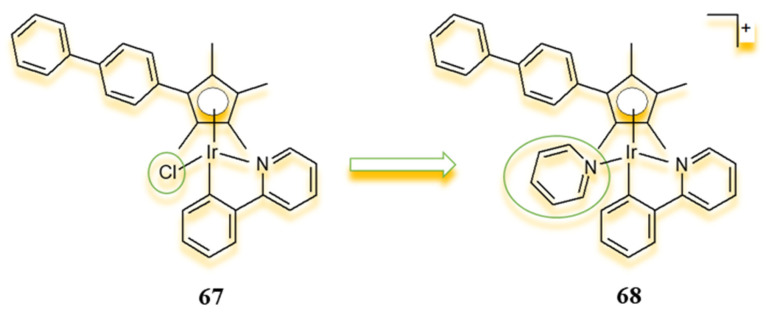
Ligand modifications replacement of the chloride anion in **67** with pyridine in **68**.

Compared with the N,N-donor analogue (IC_50_ > 100 μM), complex **66** shows significantly superior cytotoxicity (IC_50_ = 10.8 μM) (Figure 34). Furthermore, this modification of the ligands significantly increases the hydrophobicity of compounds, as shown by the value of the parameter log *p*. For complex **65** with the N,N-donor, the value of the parameter log *p* = −0.95 ± 0.06, whereas for **66** with the C,N-donor, log *p* = +1.57 ± 0.08, which results in greater cellular uptake and better anticancer activity. Studies comparing the affinity of the complexes for DNA strand bases have also been performed. The compound [(η^5^-Cp*)Ir(phpy)Cl] (**66**) binds to purines to a much greater extent [204,209]. The conversion of the chloride anion to pyridine in the half-sandwich Ir(III) complexes also increases the antitumour activity of the compounds (Figure 36).

This finding was confirmed by anticancer studies of complexes **67** and **68** against A2780 (Figure 34). We found that the complex with the pyridine ligand exhibited high antitumour activity, being approximately five times more active than its chloride analogue. In addition, further studies have shown that complex **68** is a potent inducer of ROS in A2780 cells, which is important in the search for an effective pathway to destroy cancer cells [211,212]. In addition, replacing the chloride ion with pyridine seems to be an effective strategy to prevent the deactivation of the compound, as complex **67** with the chloride ligand is rapidly hydrolysed and thus readily reacts with glutathione (GSH), presumably reducing its cytotoxicity [210]. The reported examples of half-sandwich organometallic Ir(III) compounds confirm the close relationship between the composition of complexes containing different types of ligands and the antitumor properties of the studied chemotherapeutics, and point to a number of MoA opportunities.

### 4.2. Ir(III) Complexes as Biocatalysts

**Figure 37:**
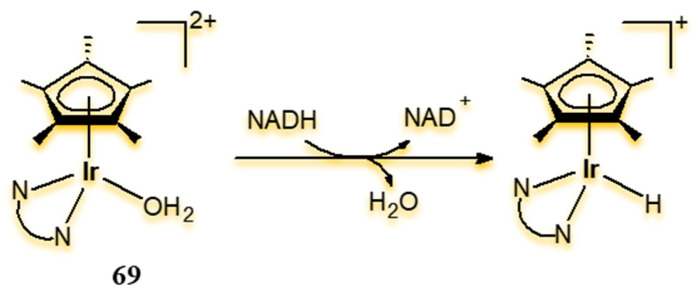
The catalytic oxidation of the NADH via Ir(III) complexes.

As mentioned above, one of the most important applications of iridium coordination complexes is their use as catalysts for many chemical reactions [213,214,215,216,217,218]. This property also suggests potential as biocatalysts for chemical transformations that occur in living organisms. Indeed, studies by P. Sadler, Z. Liu and coworkers [219] have proposed a new mechanism for catalytic quinone reduction via hydrogen anion transfer from NADH involving the piano-stool Ir(III) complex (Figure 37).

**Figure 38:**
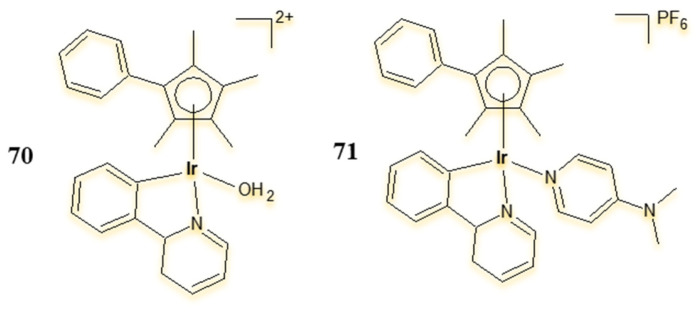
Cyclometalated iridium(III) complexes **70** and **71**.

Additional studies [220] have shown that Ir(III) cyclopentadienyl derivatives are potent catalysts for H_2_ production and can be used in ketone reduction. The above process can also be a source of active hydrogen, which reacts with oxygen to form H_2_O_2_. On this basis, researchers have proposed a new method for generating ROS in tumour cells by using organometallic compounds as intracellular oxidants [212,221]. The application of Ir(III) compounds with C,N-donor ligands may represent a new approach to cancer treatment and provide a highly effective oxidant-based therapy. On the other hand, in vivo studies have shown that both NAD^+^ and NADH play important roles as cofactors in a number of biocatalytic reactions in organisms. The most important catalysed processes include energy metabolism, antioxidation and oxidative stress, immune function and cell apoptosis [204]. These results suggest that Ir(III) compounds causing the quantitative balance of NAD^+^/NADH forms in cancer cells may be disturbed, which may indicate a novel MoA of the chemotherapeutic agents studied. Indeed, Sadler and co-authors [220], who studied an Ir(III) complex with the formula [(η^5^-C_5_Me_4_C_6_H_5_)Ir(phen)H_2_O]^2+^ (**70**) (Figure 38), reported that the compound could increase the NAD^+^/NADH ratio in A2780 cancer cells almost two-fold, presumably by transferring the hydrogen anion from NADH to biologically available substrates, thus establishing a new redox balance in cells [220].

Sadler’s team [211] also obtained new Ir(III) complex (**71**) with a C,N donor ligand (Figure 38).

The complex **71** was tested against the A2780, A549 and MCF-7, as human cancer cell lines. The results of in vitro antitumor research exposed that the **71** was highly cytotoxic against the tested cancer cells and the best for use against MCF-7 IC_50_ = 0.2 ± 0.04 μM. Its cytotoxicity was greater than that of the clinically used cisplatin. Furthermore, complex **71** was found to cause a rapid increase in ROS levels in ovarian cancer and induce mitochondrial dysfunction through the loss of MMP. These results show that Ir(III) complexes, especially those with C,N-donor ligands, have the ability to act as biological catalysts; thus, their synthesis offers the hope of obtaining chemotherapeutics with new mechanisms of action. In addition, researchers suggest that Ir(III) complexes with pro-oxidative and catalytic properties can be effectively used to address one form of drug resistance, a major clinical problem.

### 4.3. Cyclometalated Iridium(III) Complexes as Potential Sensitisers in PDT and PACT Therapy

**Scheme 4:**
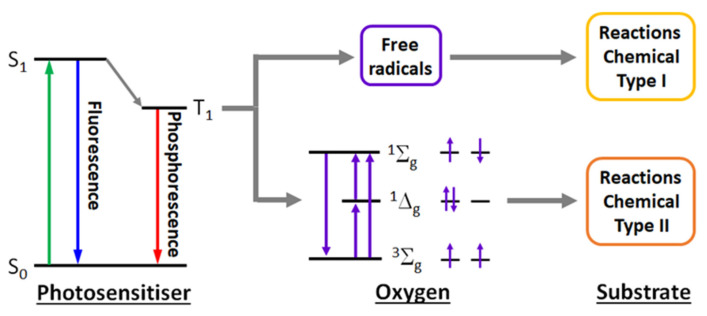
Mechanism of spectroscopic, photophysical and photochemical processes used in PDT photodynamic therapy. Self-drawn diagram based on data from the literature.

Currently, an increasing number of scientists are focusing on the interaction of Ir(III) complexes with light for the development of new, minimally invasive cancer therapies known as PDT and PACT. PDT is a non-invasive method that uses light to manage the activity of medicine in a controlled time period and in a defined space. This effect is achieved by introducing a photosensitive compound (photosensitiser, PS) into the body in the presence of oxygen (^3^O_2_). The mechanism of this method involves two types of photochemical reactions (Type I and Type II) (Scheme 4). Thanks to this method, both ^1^O_2_ and ROS quickly react with neighbouring biomolecules in cancer cells, interfering with their normal functioning and ultimately leading to apoptosis [222,223,224].

**Scheme 5:**
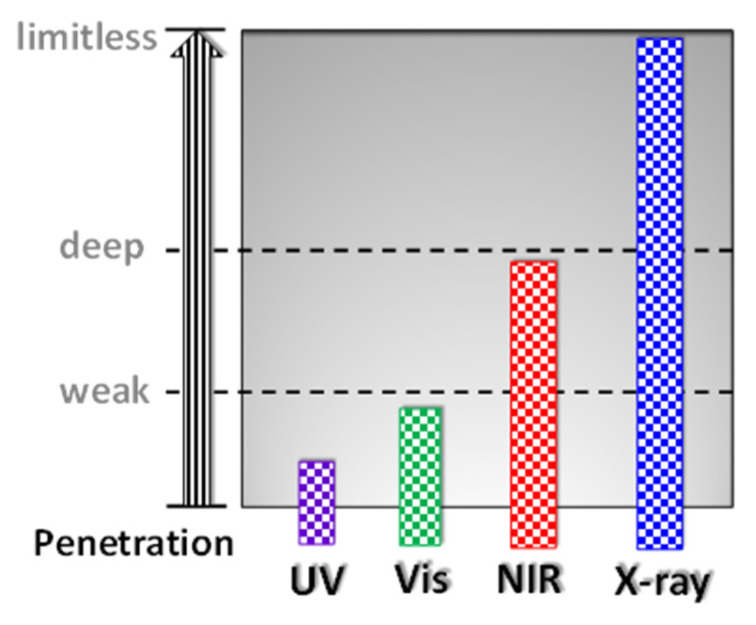
Permeability of different excitation sources through human tissue [223].

For a PS to be used clinically, it must meet two requirements: (1) strong phytotoxicity (high phototoxicity index (PI = [IC_50_]dark/[IC_50_]light), and (2) strong absorption in the so-called “phototherapeutic window” (in the range of 650–850 nm) to maximise light penetration through human tissues (Scheme 5) [225,226].

**Figure 39:**
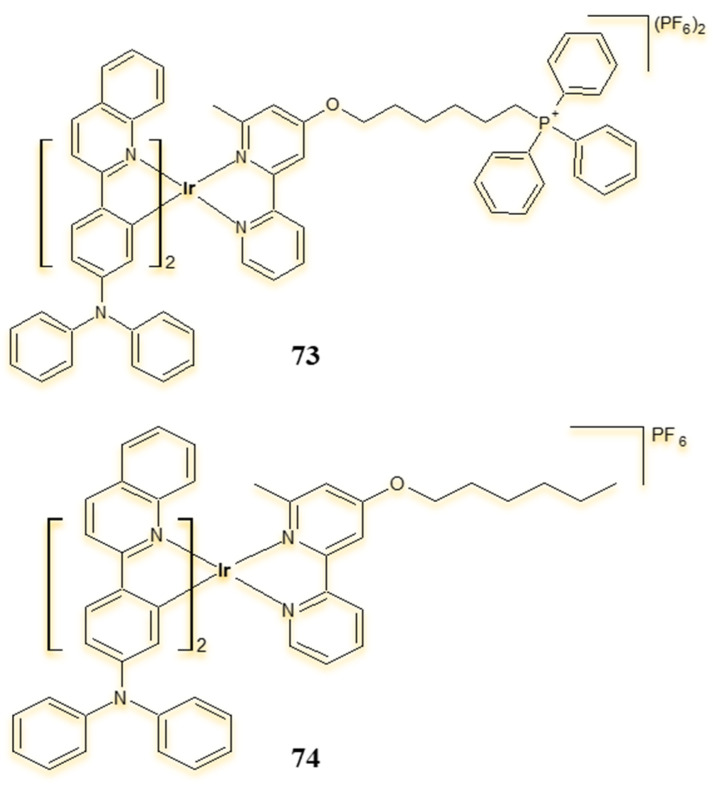
Cyclometalated iridium(III) complexes as a photosensitiser targeting mitochondria (**73**) or targeting lysosomes (**74**) in HeLa cells [236].

To date, most FDA-accepted photosensitisers used for PDT are porphyrin compounds [227,228] but they do not work very well because they do not last very long and do not make much singlet oxygen [229]. A proposed solution is the combination of (i) porphyrin and (ii) cyclometalated transition metal ions as ligand carriers. Complexes with the aforementioned ligands in which the metal has a structural function have been found to exhibit relatively long triplet state lifetimes (^3^MLCT excited state) [230,231]. The illustration of the success resulting from the use of such PSs in PDT is provided by TOOKAD (Figure S2), which completed a phase III clinical trial for the treatment of prostate cancer with success [232]. In addition, good results have been obtained with the previously mentioned Ru(II) complex TLD-1433 (Section 2.2) for the treatment of NMIBC [72] (Clinical Trials.gov Identifier NCT03053635). In this context, cyclometalated Ir(III) complexes have recently been intensively investigated as alternatives to Ru(II)-based PSs [233]. Studies have shown that these complexes have several advantages: (1) tuneable emission spectra extending into the NIR, (2) energy level regulation, (3) extensive lifetimes (1 s), (4) a high energy stabilised ligand field (ESPL), (5) the formation of excited states of ^3^MLCTs and (6) the ability to generate ROS under hypoxia via electron or energy transfer [234,235]. Excitation of a photosensitiser can be reached via the absorption of a single-photon PDT or two-photon PDT. The total energy required to induce emission is split equally between the two. Multiphoton excitation of metal complexes has the advantage of extending the excitation wavelength of the metal complex [90]. Researchers working on the use of Ir(III) complexes as photosensitisers have addressed a number of additional issues, including investigating the effect of the location of a chemotherapeutic agent within a cell on its therapeutic effect. To this end, Zhao and Huang [236] designed and synthesised two Ir(III) photosensitisers with C,N-donor ligands that (a) specifically target mitochondria and (b) target lysosomes in HeLa cells (endometrial cancer adenocarcinoma) (Figure 39).

**Figure 40:**
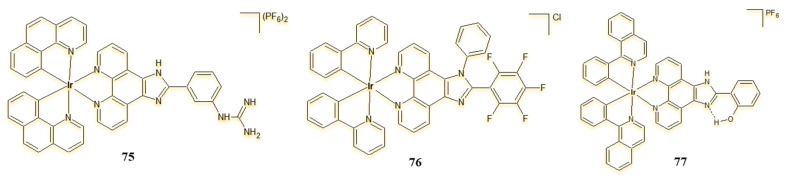
Cyclometalated iridium(III) complex containing guanidine as a ligand **75** [239] and **76** [240] and **77** [241].

The therapeutic efficiency of PDT is usually limited by low oxygen concentrations in solid tumours (4%) [237], and researchers have evaluated the phototoxicity of complexes **73** and **74** (Figure 39) under normoxia and hypoxia. Compound **73** exhibited excellent light-activated cytotoxicity, with minimal dark toxicity, making it a promising candidate for photodynamic therapy, potentially even in hypoxic tumour environments where traditional PDT is less effective. Conversely, the presence of **74** (which targets lysosomes) resulted in cell proliferation rates exceeding 66% under both conditions. The different results were explained by the authors as a result of the different localisation of the complexes. The presence of PS in the mitochondria suppresses mitochondrial respiration, leading to elevated oxygen levels in the mitochondria, thereby promoting PDT. Recent studies have shown that PSs located in lysosomes are also pharmacologically attractive for the selective destruction of cancer cells, but they are sensitive to pH changes [238]. Studies performed with complex **75** (Figure 40), showed moderate cytotoxicity against the following cancer cells: HeLa (IC_50_ = 8.96 ± 0.34 μM), HepG2 (IC_50_ = 20.35 ± 0.83 μM), MCF-7 (IC_50_ = 13.44 ± 0.53 μM), CNE-2 (IC_50_ = 44.99 ± 4.27 μM) and A549 (IC_50_ = 13.65 ± 0.63 μM). Curiously, this complex, which specifically targets mitochondria, was more active than cisplatin against cisplatin-resistant A549R (IC_50_ = 13.63 ± 2.25 μM) cells [239].

Further studies showed that this complex stopped the cell cycle at the G0/G1 phase and caused cancer cells to die via pathways requiring ROS. In contrast, Chao and coworkers [240] obtained cyclometalated Ir(III) complexes with an N,N-donor ligand with varying numbers of substituted fluorine atoms. The complex **76** which has the highest number of fluorine atoms, was characterised by superior cytotoxicity and selectivity towards diseased cells, as well as by marked effects on the A549R (IC_50_ = 0.7 ± 0.2 μM) cells [240].

**Figure 41:**
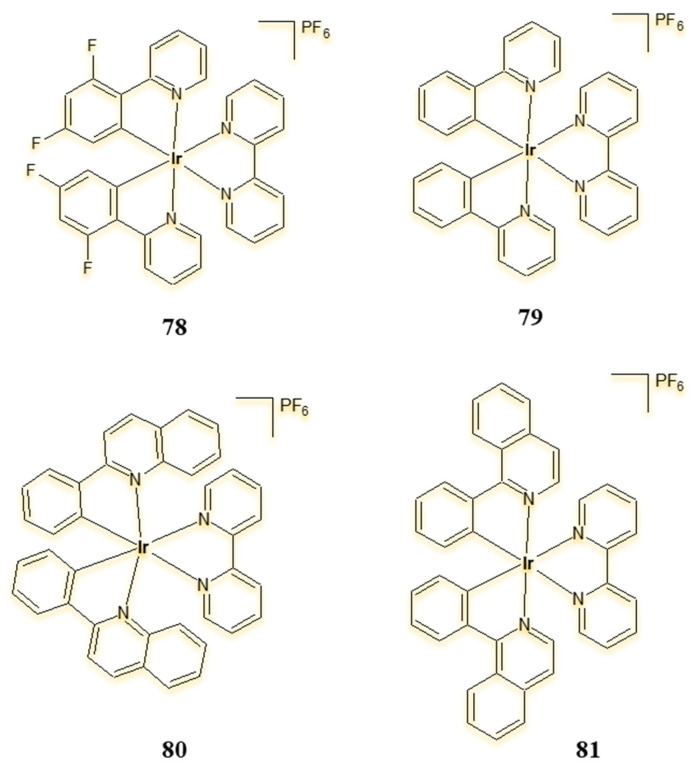
Cyclometalated iridium(III) complexes as potential PS [242].

Further studies on the MoA of **76** in question (Figure 40) revealed that the complex acts by depolarising MMP and activating caspases, causing the apoptosis of diseased cells via mitochondrial pathways. In contrast, studies by P. Gupta and coworkers [241] on a series of Ir(III) complexes with C,N donor ligands, of which the complex (Figure 40 is the most notable, show different localisations of the test compounds in the cell. The strong intramolecular interactions via hydrogen bonds (O-H···N) occurring in the complex in question (Figure 40) were found to be responsible for the specific localisation of the compound in the ER of the cells. The death of almost all the cells was detected after 1 h of light exposure. Like the Gupta team, T. H. Kwon and co-authors [242] reported localisation in the ER for the resulting group of photosensitisers based on Ir(III) complexes **78**–**81**. The coordination compounds differed in terms of the nature of the ligands (Figure 41).

**Figure 42:**
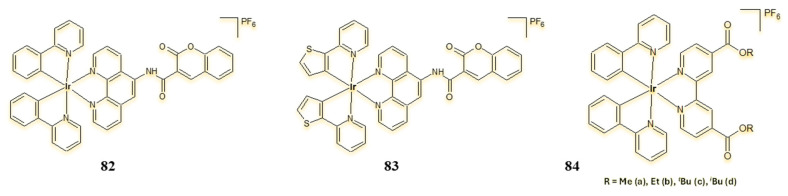
Cationic Ir(III) compounds **82**, **83** [246] and ester-modified Ir(III) complexes **84** [245].

The compounds effectively induced the death of cancer cells SKOV-3 and MCF-7 by generating ROS after 10 s of light irradiation. The authors showed that the likely MoA results from the cross-linking and oxidation of proteins in cancer cells located near the ER and the mitochondrion. Recently, an increasing number of researchers turned their attention to developing therapeutic agents that target mitochondria and that also possess luminescent properties [242,243,244,245,246], which offer the possibility of monitoring the therapeutic effect in situ. In addition, mitochondria-targeting compounds may be a strategy to combat platinum-resistant cancers. Mitochondria are organelles that are vital for cellular energy production and are implicated in many cellular activities, including the generation of ROS, which can result in cell death. Mao and coworkers [242,243,244,245,246], among others, have obtained several series of phosphorylating complexes with targeted mitochondrial localisation. Complexes **82** and **83** (Figure 42) show “theranostic” functions (with both therapeutic and diagnostic functions) [246].

In addition, detailed transcriptional and genome-wide studies have revealed a link between cytotoxicity and pathways associated with mitochondrial dysfunction, which can lead to cell apoptosis [246]. Further studies by Mao’s team [245] led to cyclometalated ester-modified Ir(III) complexes **84**. These compounds initiate ATP depletion, loss of MMP and elevation of ROS levels, resulting in the induction of apoptosis.

**Figure 43:**
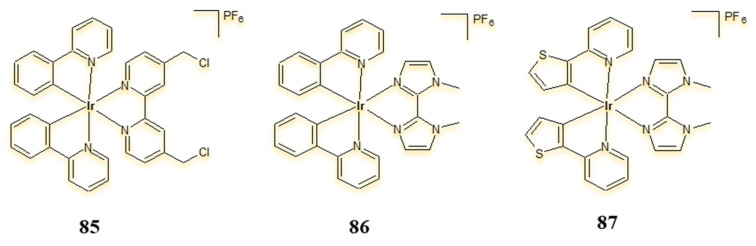
Cyclometalated Ir(III) complexes **85**–**87** [243,247].

Furthermore, a study using cyclometalated complexes to establish cellular localisation revealed that compounds **85**–**87** all localised to the mitochondria of A549 cell lines (Figure 43) [243,247].

**Figure 44:**
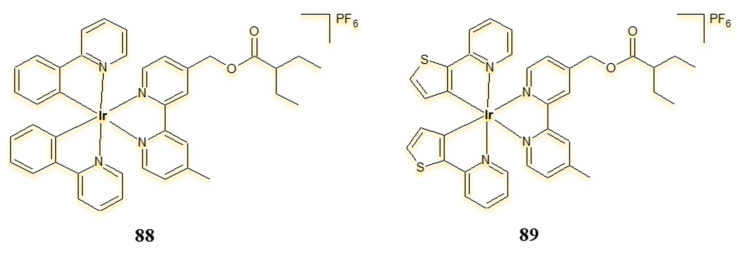
Cyclometalated Ir(III) complexes **88** and **89** [248].

Interestingly, the phosphorescent cyclometalated complex **85** could also be used to track mitochondrial morphological changes, allowing insight into their anticancer mechanisms. Furthermore, Ir(III) complexes **86** and **87** cause MMP depolarisation, cell cycle ATP depletion, mitochondrial metabolic dysfunction and the induction of OS [247]. In addition, Mao and coworkers [248] investigated phosphorescent **88** and **89** compounds (Figure 44), which exhibit significantly greater antiproliferative activity, including in A549R cells.

Furthermore, these compounds generate a series of mitochondrial disfunctions in HeLa cells, as follows: MMP depolarisation, ROS generation, cell cycle arrest and caspase triggering, leading to apoptosis.

**Figure 45:**
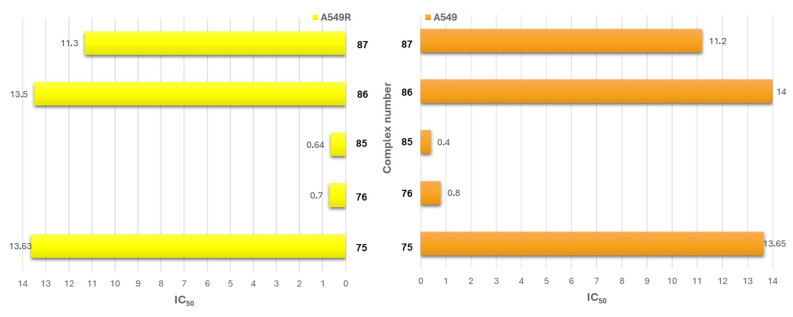
Visual representation of cytotoxic activity of Ir(III) complexes with selected ligands against A549 and A549R cells (IC_50_).

In order to illustrate the influence of similar ligands on the cytotoxic properties of Ir(III) complexes, IC_50_ values were compared for the following cancer cell lines: A549 and A549R (Figure 45).

**Figure 46:**
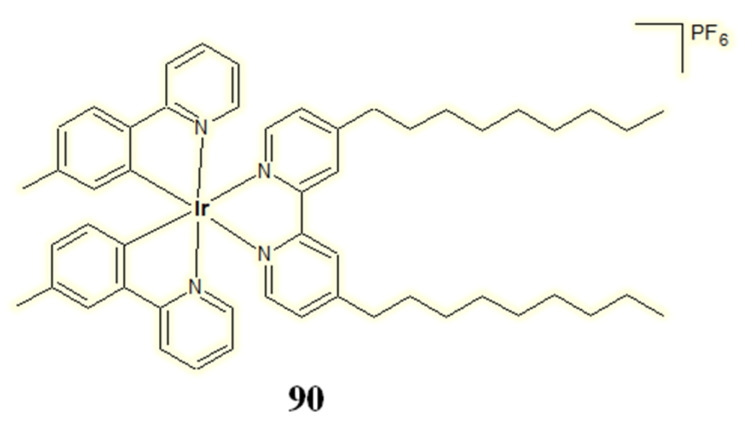
Cyclometalated Ir(III) complex as H-Ras/Raf-1 inhibitor [249].

Of particular note are the studies of D.-L. Ma, Ch.-H. Leung and team [249], who obtained an Ir(III) complex containing an N,N-donor and two C,N-donor ligands (Figure 46).

The compound, especially its D-enantiomer, was found to be distinguished by its inhibitory properties against H-Ras/Raf-1 and its downstream pathways both in vitro and in vivo. The elevated activity of the aforementioned enzymes increases tumour initiation, progression and metastasis (renal cell carcinoma) [250]. It is noteworthy that the compound showed no toxic effects on the healthy cells of the organism.

**Figure 47:**
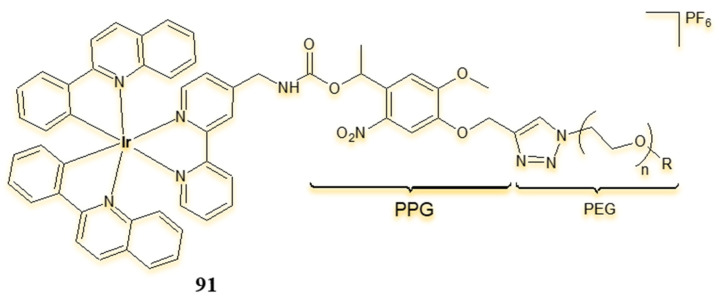
Cyclometalated Ir(III) complex with potential application in PACT [251].

As mentioned previously in the discussion of ruthenium complexes (Section 2.2), a new way of treating of oxygen-deprived tumours is photoactivated chemotherapy (PACT). This method photoactivates compounds using various mechanisms, eliminating the need for oxygen in the cell and offering control of chemotherapeutic activity. As an alternative to studies using Ru(II) complexes in the PACT method, Lo’s team presented studies using the photoactive Ir(III) complex (Figure 47) [251].

Similarly to the studies on Re(I) [252] and Ru(II) [253] complexes, Lo and co-authors attached a photolabile nitrobenzyl protecting group (PPG) and a polyethylene glycol (PEG) molecule to the ligand for the initial photostabilisation of the compound prior to irradiation, increasing the water solubility of the compound. After light activation, the complex showed higher cytotoxicity against tumour-bearing HeLa cells than it did under dark conditions. Furthermore, the authors showed that the studied complex localises to the mitochondria and that its phototoxicity is not due to ROS generation but rather to the photorelease of PPG-PEG, which allows for oxygen-independent photocontrol of the compound’s cytotoxicity.

## 5. Conclusions

This review article presents a comprehensive analysis of the literature explaining the global involvement of research teams in solving an important social problem, namely the search for effective metal-ion-based anticancer drugs, namely ruthenium, rhodium and iridium, as a promising alternative to platinum drugs, since a serious problem with their resistance has been identified in clinical treatment.

In summary, based on the existing literature, the objective was to highlight the most significant achievements made by scientists in the search for new chemotherapeutics that have entered the clinical trial phase. The analysis of ruthenium coordination compounds presented in this paper demonstrated that metal ions contained in complexes have the capacity to participate in biological redox reactions. Furthermore, ruthenium, which has the ability to alter its oxidation state under physiological conditions (Ru(III)/Ru(II)), offers a multitude of possibilities for the strategic design of new chemotherapeutics. It is noteworthy that the research achievements of Keppler’s team are particularly significant in this context. The team’s research proved that potential Ru(III)-based chemotherapeutics can be used as prodrugs in cancer therapy. Specifically, NKP-1339 is a complex that has undergone clinical testing (phase I) and has demonstrated encouraging results in the inhibition of the growth of solid tumours, including non-small cell lung cancer, colon cancer and, most notably, neuroendocrine tumours of the gastrointestinal tract.

Furthermore, in connection with the intensive development of non-invasive photodynamic therapy (PDT), the research conducted by S. A. McFarland and others, which led to the discovery of the Ru(II) TLD-1433 complex, is a noteworthy achievement. This compound is the only Ru(II) complex for which phase II clinical trials have been conducted in the treatment of patients with carcinoma in situ (CIS) that is insensitive to bacillus Calmette-Guérin (BCG), and with non-invasive bladder cancer (NMIBC).

It should be noted that intensive research is also being conducted that is searching for Ru(II) and Ir(III) complexes that can be used as PS not only in PDT, but also in PACT. Namely, as a result of the dissociation of labile ligands of the complex under the influence of light, the potential chemotherapeutic agent exhibits a dual (multitarget) effect: as an enzyme inhibitor (ligand) and as capable of direct interaction with DNA (metal ion). In the current paradigm, multitarget compounds are considered to be more effective thetremlettrapeutic agents in overcoming cell resistance mechanisms.

The findings of studies conducted on a group of Ir(III) and Ru(II) half-sandwich complexes are also noteworthy. The pioneering contribution of the Dyson and Sadler teams to the development of this topic should be particularly emphasised [2,100]. It has been found that some complexes exhibit DNA-binding properties, while others disrupt the redox balance in cells, as evidenced by their ability to biocatalyze. Iridium(III) complexes have been shown to have the ability to influence the redox balance, causing a change in the NAD+/NADH ratio in cancer cells. This discovery suggests the possibility of a new mechanism of action for Ir(III)-based chemotherapeutics. In addition, the researchers synthesised the ruthenium compounds RAPTA-C and RAED, which are currently being researched in preclinical trials.

However, the presented studies on Rh(III) complexes have shown that the metal ion can play a dual role: a structural role or as a biocatalyst. Octahedral rhodium(III) complexes act as a factor modelling the activity of selected enzymes. Among other things, it has been shown that the octahedral configuration is consistent with the binding pocket model of Src kinase, thus increasing the ability to inhibit Src protein tyrosine kinase (complex no. **25**). Based on the geometric effect, rhodium inhibitors of the NEDD8 enzyme (an enzyme that controls the activity of ubiquitin ligase) have also been developed (complex no. **26**).

A review of research on rhodium and iridium chemotherapeutics indicates the important role of rhodium and iridium ions, next to ligands, in coordination compounds with regard to their biological properties. Studies have shown that complexes of this type may be a promising alternative to platinum drugs, because they may also exhibit other mechanisms of action (MoA). Table 1 presents significant differences in the mechanisms of action, pharmacokinetics and toxicity profiles of rhodium and iridium complexes.

Thus, rhodium and iridium complexes provide a novel approach to treating cancer by modifying redox and metabolic processes within cancer cells. By contrast, the established mechanism of action of platinum chemotherapeutics is the covalent binding of a Pt complex to DNA, or non-covalent interactions such as intercalation or groove binding. At the same time, clinical studies have identified platinum drug resistance as a problem in patients with recurrent cancer. However, a review of the literature relevant to this work has shown that, despite intensive research on chemotherapeutics based on Ru, Rh and Ir elements, only a few complexes have been selected for advanced clinical trials. The reason for this is believed to lie in certain properties of organometallic complexes, which constitute significant limitations when it comes to classifying a given compound as a drug. These properties include the following: (i) limited solubility in an aqueous environment, which hinders penetration through cell membranes; (ii) a lack of a specific means to transport the complexes through cell membranes; and (iii) the redox instability of the complexes under the conditions of the conducted studies. Therefore, scientists face challenges in solving the above problems.

It is predicted that the intensive research conducted by scientists on the coordination compounds of the aforementioned metals, coupled with a substantial number of positive research results, will cause the development of new theranostic drugs in the near future. These new drugs will enable effective cancer therapy and provide an alternative to treatment with the platinum-based drugs used to date.

## Figures and Tables

**Table 1:** Comparative characteristics of selected biological properties of rhodium and iridium potential chemotherapeutics.

Criterion	Rhodium (Rh) Complexes	Iridium (Ir) Complexes
Mechanism of action	metabolic enzyme inhibitors (**25**–**28**, Figure 14 and Figure 15; **46a**–**e**, Figure 22) catalytic modulation of redox processes in cellular metabolism (**34**–**37**, Figure 18) non-covalent (**47a**–**e**, Figure 23; **49**, Figure 25; **53a**–**c**, Figure 28) and covalent interaction with DNA (**48**, Figure 24; **55**, Figure 29)	disruption of mitochondrial function, ROS generation (**68**, Figure 36; **71**, Figure 38; **76**, Figure 40; **78**–**81**, Figure 41; **84**–**89**, Figure 42, Figure 43 and Figure 44) possibility of photoactivation as photosensitizers in PDT (**73**–**75**, Figure 39 and Figure 40; **78**–**81**, Figure 41)/PACT (**91**, Figure 47) biocatalysts in the catalysis of chemical transformations (**69**, Figure 37; **70**, Figure 38) non-covalent interaction with DNA (**62**–**64**, Figure 33; **65, 66**, Figure 35) theranostics (**82**, **83**, Figure 42)
Selectivity	High (catalytic activation possible in the tumour environment)	High in the case of photoactivation; moderate without it
Pharmacokinetics	High stability in plasma; possible binding to transferrin; active transport to cells	High lipophilicity, and accumulation in mitochondria and liver; slow elimination
Toxicity	Low; possible mild metabolic disturbances and oxidative stress	Potential hepatotoxicity and mitochondrial toxicity (dose-dependent and light-dependent)
Stage of research	Preclinical; several compounds in early biological testing	Preclinical; in vitro and in vivo testing, PDT/PACT concept development and bioimaging
Therapeutic potential	Very high—due to new MoA and lower toxicity	High—possibility of photocontrol and selectivity

## Data Availability

No new data were created or analyzed in this study. Data sharing is not applicable to this article.

## Supplementary Materials

The following supporting information can be downloaded at: https://www.mdpi.com/article/10.3390/ph18111728/s1, Figure S1. Structural formula of complexes (where **59a** R = CH_2_Ph; **b** R = CH_2_CH_2_Ph; **c** R = CH_2_C_6_H_4_-p-tBu) [200]; Figure S2. Structure of the palladium complex—TOOKAD [72].

## Abbreviations

PSs = photosensitisers, PDT = photodynamic therapy, PACT = photoactivated chemotherapy, HL-60 = human leukaemia, SKMEL28 = human melanoma, A2780 = ovarian cancer, A2780cisR = cisplatin-resistant ovarian cancer, HeLa = cervical cancer, MB-MDA-231 = metastatic large breast cancer, CHO = noncancerous ovarian, BGM = renal epithelial cell line, MCF-7 = breast cancer cells, HCT-116 = colon cancer, MRC-5 = noncancerous human lung fibroblasts, HT-29 = colon cancer, MIAPaCa-2 = pancreas, COLO-316 = ovarian cancer, Hs-27 = human skin fibroblasts, NCI-H460 = non-small cell lung cancer, SiHa = cervix cancer, SW480 = colon cancer, HEK-293 = human embryonic kidney cells, A549 = lung cancer, A549R = cisplatin-resistant cells, ER = endoplasmic reticulum, SKOV-3 = ovarian cancer, ROS = reactive oxygen species, MoA = mechanism of action, ^1^O_2_ = singlet oxygen, UPS = ubiquitin–proteasome system, Hs-27–humam skin fibroblasts, B16F10 = murine melanoma, THP-1 = human monocytic leukaemia overian, NMIBC-noninvasive bladder cancer, LC_50_ = concentration re-quired to kill 50% of the cells, FDA = Food and Drug Administration, NIR = near-infrared, MMP = mitochondrial membrane potential, OS = oxidative stress.
