# Supplementary material for: Review of the Most Important Research Trends in Potential Chemotherapeutics Based on Coordination Compounds of Ruthenium, Rhodium and Iridium
Source: Pharmaceuticals (Basel). 2025 Nov 13;18(11):1728. doi: 10.3390/ph18111728 (PMC12655325; doi:10.3390/ph18111728)
Supplement: Supplementary file 1 [file pharmaceuticals-18-01728-s001.zip › pharmaceuticals-3930517-supplementary.pdf]

Supplementary Information

Review of the most important research trends in potential chemotherapeutics based on coordination compounds of ruthenium, rhodium and iridium

Agnieszka Gilewska, Barbara Barszcz, Joanna Masternak**

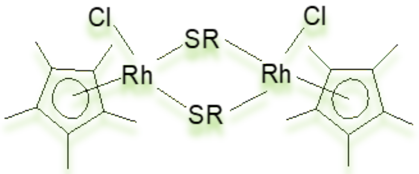

59

Figure S1. Structural formula of complexes (where **59a** R = CH_2Ph; **b** R = CH_2CH_2Ph; **c** R = $CH_2C_6H_4$-p-tBu) [200].

72

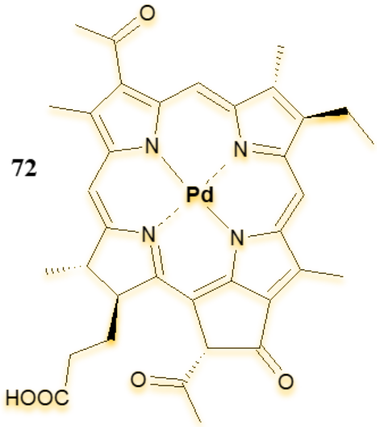

Figure S2. Structure of the palladium complex – TOOKAD [72].
